# Long-Term Dietary Consumption of Grapes Affects Kidney Health in C57BL/6J Mice

**DOI:** 10.3390/nu16142309

**Published:** 2024-07-18

**Authors:** Asim Dave, Eun-Jung Park, Paulette Kofsky, Alexandre Dufresne, Soma Chakraborty, John M. Pezzuto

**Affiliations:** 1 Department of Biology, Center for Computational and Integrative Biology, Rutgers University, Camden, NJ 08102, USA; davea3@mskcc.org; 2 Immunology Program, Memorial Sloan Kettering Cancer Center, New York, NY 10065, USA; 3 College of Pharmacy and Health Sciences, Western New England University, Springfield, MA 01119, USA; eunjung.park@wne.edu (E.-J.P.); paulette.kofsky@wne.edu (P.K.); 4 Baystate Research Facility, Baystate Medical Center, Springfield, MA 01199, USA; alexandre.dufresne@baystatehealth.org; 5 Department of Pathology, UMass Chan Medical School-Baystate, Springfield, MA 01199, USA; smcshoma@gmail.com; 6 Department of Medicine, UMass Chan Medical School-Baystate, Springfield, MA 01199, USA

**Keywords:** RNA-Seq, renal function, pathway analysis, kidney phenotype, gender differentiation

## Abstract

Starting at 4 weeks of age, male and female C57BL/6J mice were provided with a semi-synthetic diet for a period of one year and then continued on the semi-synthetic diet with or without grape supplementation for the duration of their lives. During the course of the study, no variation of body weights was noted between the groups. At 2.5 years of age, the body-weight-to-tissue-weight ratios did not vary for the liver, colon, muscle, prostate, or ovary. However, relative to the standard diet, the body/kidney weight ratio was significantly lower in the male and female groups with grape-supplemented diets. With the mice provided with the standard diet, the BUN/creatinine ratios were 125 and 152 for males and females, respectively, and reduced to 63.7 and 40.4, respectively, when provided with the grape diet. A histological evaluation suggested that this may be due to enhanced/improved perfusion in the kidney as a preventive/protective effect. In response to the dietary grapes, an RNA seq analysis revealed up-regulation of 21 and 109 genes with male and female mice, respectively, with a corresponding down-regulation of 108 and 65 genes. The downward movement of the FPKM values in the males (*alox5*, *btk*, *fga*, *fpr1*, *hmox1*, *lox*, *ltf*, *lyve1*, *marco*, *mmp8*, *prg4*, *s100a8/9*, *serpina3n*, and *vsig4*) and upward movement of the FPKM values in the females (*camp*, *cd300lf*, *cd72*, *fcgr4*, *fgr*, *fpr2*, *htra4*, *il10*, *lilrb4b*, *lipg*, *pilra*, and *tlr8*) suggest beneficial kidney effects. The expression of some genes related to the immunological activity was also modulated by the grape diet, mainly downward in the males and upward in the females. The reactome pathway analysis, KEGG analysis, and GSEA normalized enrichment scores illustrate that several pathways related to immune function, collagenase degradation, extracellular matrix regulation, metabolism of vitamins and cofactors, pancreatic secretion, aging, and mitochondrial function were enriched in both the males and females provided with the grape diet. Overall, these results indicate that the long-term dietary consumption of grapes contributes to renal health and resilience against fibrosis and related pathologies.

## 1. Introduction

The classical concept of nutrition is the consumption of foods and beverages that enable our bodies to grow, reproduce, and fundamentally thrive. This is self-evident considering, for example, the multi-year transfiguration of a fetus to an adult human being. Certainly, malnutrition may lead to consequences such as tiredness, disease, organ failure, cognitive deficit, cachexia, and death.

In addition, however, over the past few decades, the broad-based influence of diet on health and well-being has been emphasized. In a study that followed 66,719 women from the Nurses’ Health Study and 42,016 men from the Health Professionals Follow-up Study, a lower risk of overall death and from dying of cancer, cardiovascular disease, and respiratory disease was reported [1]. Risk reduction plateaued with the daily consumption of about five servings of fruit and vegetables, which is the general dietary recommendation provided by organizations such as the American Health Association and the National Cancer Institute. Rather than, or in addition to, the nutritive value of fruits and vegetables, these types of health benefits are associated with the ingestion of phytochemical constituents. A large number of such components have been identified [2], and a myriad of studies have reported potential mechanisms of action [3].

In the realm of fruits and vegetables, our work has largely focused on grapes. In the context of health, grapes garnered a great deal of attention due to the relatively unique presence of resveratrol. Following a report describing the cancer chemopreventive potential of resveratrol [4], thousands of manuscripts have appeared investigating the potential to prevent or treat a broad array of disease states [5]. It should be noted, however, that the quantity of resveratrol present in a normal diet is miniscule compared to the doses utilized in studies conducted with laboratory animals or human clinical trials. In essence, resveratrol has evolved and continues to be studied as a dietary supplement or natural product drug. Most would agree that not much response would be anticipated from normal dietary intake.

As a whole food, the grape contains hundreds of phytochemical constituents [6] in addition to resveratrol. In the context of diets, if grapes were to induce a physiological response, it would be anticipated that this conglomeration of components would be responsible rather than a single component. Hinging on this concept, utilizing a standardized surrogate representative of the whole grape, numerous studies have been performed to assess the possible influence of grapes on health. Interestingly, effects have been observed that are relevant to a number of human conditions involving the heart, gastrointestinal, bladder, cognition, vision, skin, etc. [7]. Various mechanisms may apply, including the modulation of the human microbiome [8,9].

A response mechanism of particular interest to us relates to the field of nutrigenomics, especially the potential of diets to modulate phenotypic expression. Employing whole grapes as a prototype, dietary consumption in mouse models alters genetic expression in various organs [10,11]. Further, when given concomitantly with a high-fat diet, grape consumption improves cognition [12], retards fatty liver development [13], and enhances lifespan [13]. Notably, changes in genetic expression correlate with the alteration of the metabolome [14].

In the current work, rather than dealing with the amelioration of adverse effects resulting from the consumption of a high-fat diet, for example, we were interested in exploring how long-term grape consumption might alter the parameters associated with a “normal” life- and health-span. After providing male and female mice with a semi-synthetic standard diet (SD) for a period of one year, they were divided into four groups, half of which continued receiving the standard diet and half of which received the standard diet supplemented with grapes (SDG). After an additional 1.5 years, the animals were sacrificed for observation. Surprisingly, the most profound overt effect was on the kidney. This led us to perform more in-depth analysis of the organ. The results are reported here.

## 2. Results

### 2.1. Effect of Diets on Mouse Body Weights and Tissues

#### 2.1.1. Effect of Diets on Mouse Body Weights

**Figure 1:**
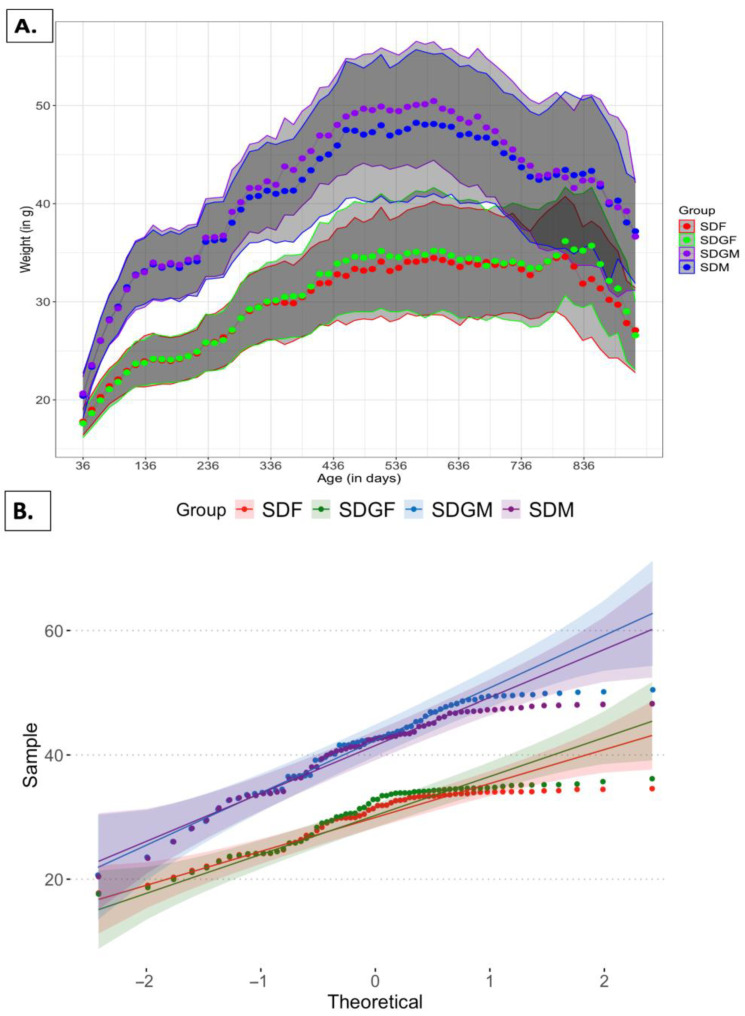
Body weight of the mice. (**A**) Body weight of SDM, SDGM, SDF, and SDGF groups. Data points indicate the average of the respective groups, and the shaded areas encompass standard deviation. (**B**) Distribution of SDM, SDGM, SDF, and SDGF groups represented through a normal distribution (Theoretical units = deviations in grams; sample units = counts/number of samples).

Figure 1 illustrates the body weight of the four groups of mice for the duration of the study period (2.5 y). As expected, the average body weight of males was greater than females, but within groups of the same gender, i.e., SDM vs. SDGM and SDF vs. SDGF, there was no significant difference. Specifically, ANOVA revealed a significant difference in the weight between the SDM, SDGM, SDF, and SDGF groups [F(3, 253) = 74.03, *p* < 0.001]. Tukey’s HSD post hoc test showed significant differences in the weight between SDGM and SDF (*p* < 0.001), SDM and SDF (*p* < 0.001), SDGM and SDGF (*p* < 0.001), and SDM and SDGF (*p* < 0.001). There was no significant difference in the weight between the SDM and SDGM groups (*p* = 0.857) or between the SDF and SDGF groups (*p* = 0.941).

The lack of difference between the respective gender groups is further supported by the scatter plot presented in Figure 1B. This plot illustrates the frequency distribution of individual mouse weights and their deviation from the mean value for each dietary group. The mean weights for SDM, SDGM, SDF, and SDGF were 40.76 ± 6.62, 41.59 ± 7.23, 29.80 ± 4.57 and 30.40 ± 5.03 g, respectively. In sum, these data indicate that the introduction of a grape-enriched diet does not lead to significant alterations in body weight compared to a standard diet in both male and female mice.

#### 2.1.2. Effect of Diets on Mouse Tissues

After being maintained on a standard diet for 1 year, followed by a standard diet supplemented with 5% grape powder for an additional 1.5 years, five animals from each group were randomly selected for more detailed analyses. As summarized in Table 1, the weight of the liver, colon, kidney, muscle, prostate, or ovary did not significantly vary between SDM vs. SDGM, or SDF vs. SDGF, nor did colon length. Tissue to body weight ratios were more revealing. Again, among the respective groups, no variation was noted with the liver, colon, muscle, prostate, or ovary. However, in the case of both male and female mice, the kidney/body weight ratio was significantly lower in the groups that were provided with the grape-supplemented diet.

### 2.2. Evaluation of Blood Chemistry and Histology

As summarized in Table 2, analysis of blood chemistry revealed no statistically significant differences among the parameters examined (aside from a slight change of the Na level in female mice), although it was noted that the average BUN levels in both of the standard diet groups (male and female) exceeded the reference values.

**Figure 2:**
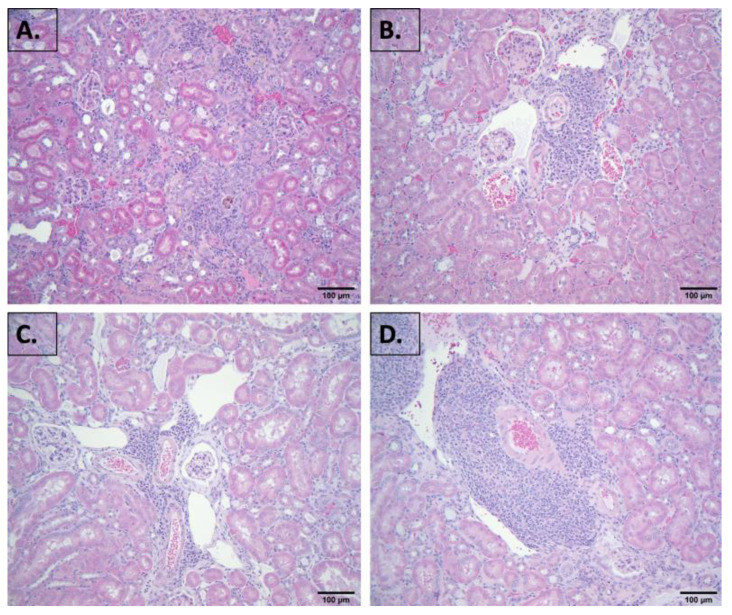
Representative photomicrographs of the kidney from (**A**) male, SD, (**B**) male, SDG, (**C**) female, SD, and (**D**) female, SDG. Tissues were processed, and slides were prepared as described in “Materials and Methods”. Overall, male and female mice given the standard diet predominantly showed chronic interstitial inflammation, compared to mice given grape-supplemented diet, showing perivascular inflammation. Four-micron sections were stained with H & E. Original magnification, 20×.

A histopathological evaluation of the liver, colon, muscle, prostate, or ovary from all of the designated groups, using both H&E and Masson’s trichrome stains, did not reveal any profound differences or a common pathology. Similarly, a histopathological evaluation of the kidney did not reveal profound differences between the groups (Figure 2). Overall, male and female mice given the standard diet predominantly showed chronic interstitial inflammation, compared to mice given the grape-supplemented diet, showing perivascular inflammation.

### 2.3. Survival Analysis

**Figure 3:**
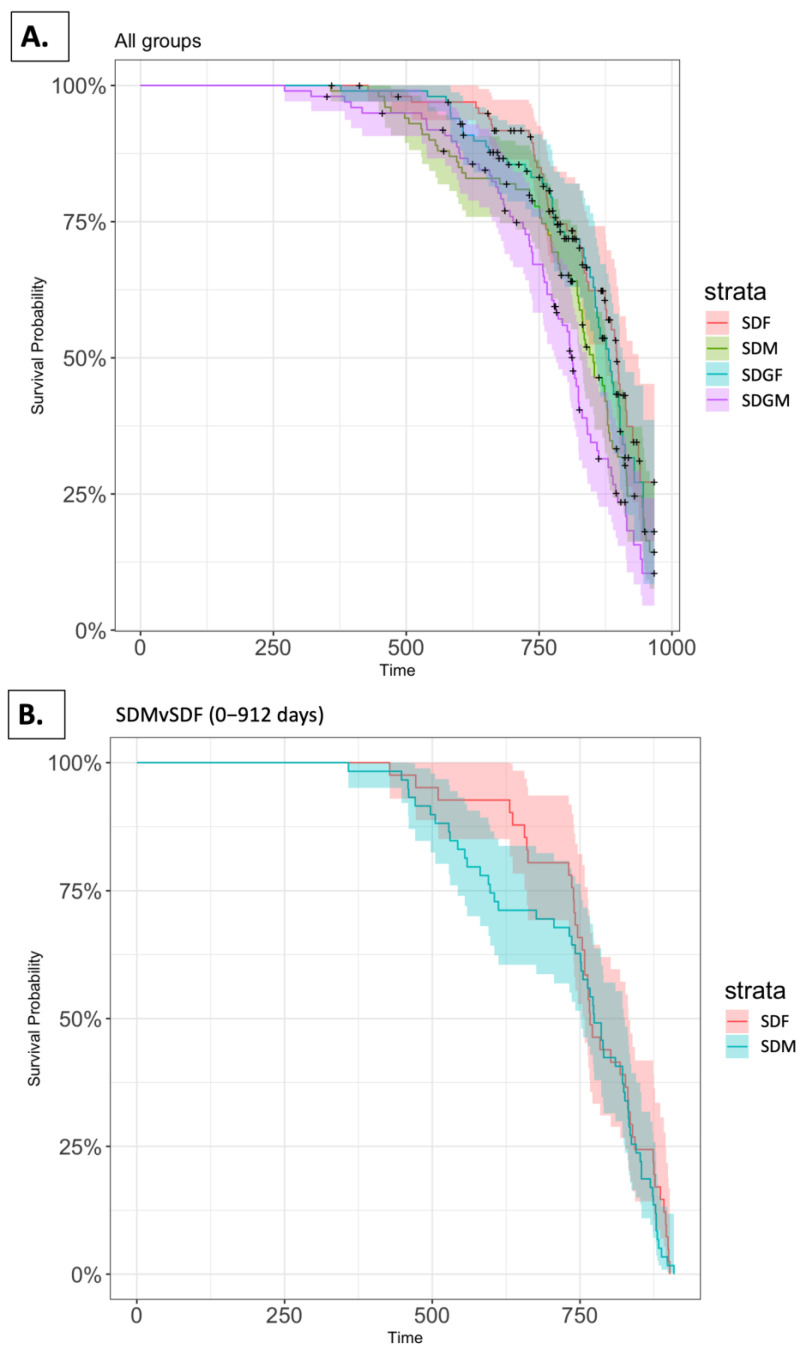

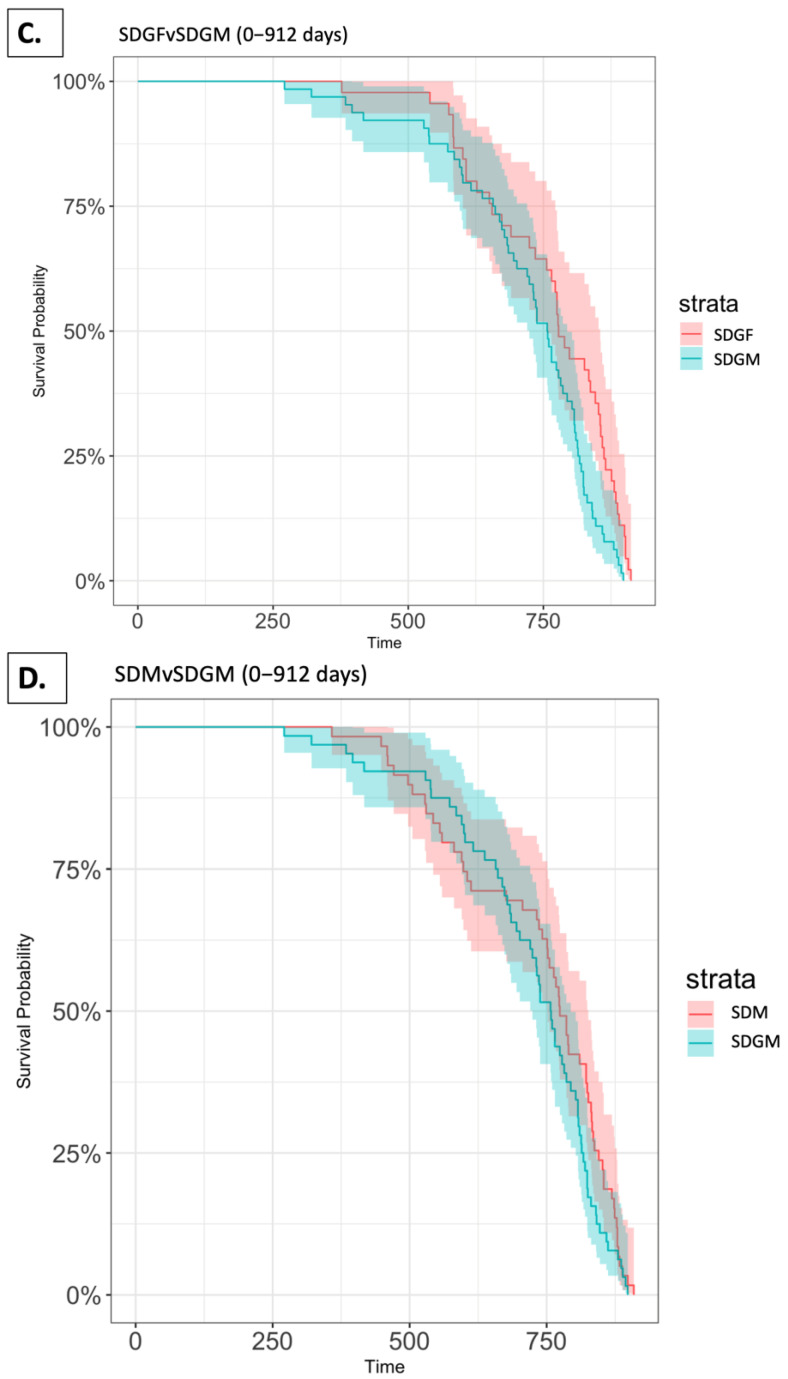

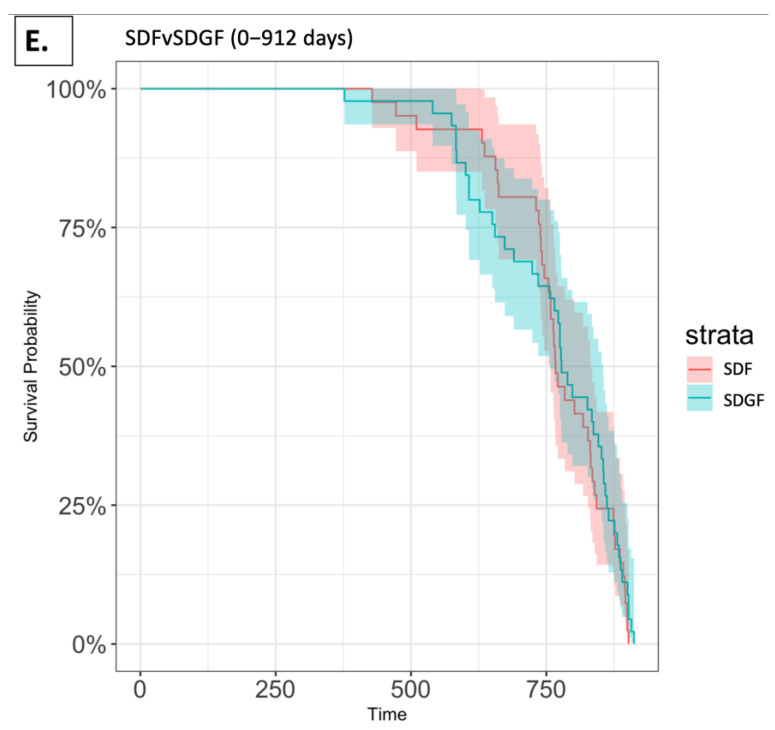
Kaplan–Meier plots showing the survival of mice in SD, SDGM, SDF, and SDGF. Kaplan–Meier plot for (**A**) lifetime comparison of SDM, SDF, SDGM, and SDGF. (**B**) SDM and SDF for 912 days. (**C**) SDGM and SDGF for 912 days. (**D**) SDM and SDGM for 912 days. (**E**) SDF and SDGF for 912 days. The shaded regions in all panels show the confidence interval, which is 0.95.

Survival of the mice including in the SDM, SDF, SDGM, and SDGF groups from the start of the study until the end of life is illustrated in Figure 3A. Comparison of Kaplan–Meier plots for SDM (observed events—67), SDF (observed events—46), SDGM (observed events—69), and SDGF (observed events—48) indicated a statistically significant difference when comparing the four groups as a whole (*p* < 0.001, log-rank test). [(O-E)^2^/E and (O-E)^2^/V represent contributions to the Chi-square statistic for the log-rank test (E, expected value; O, observed value; V, variance). For SDM, (O-E)^2^/E and (O-E)^2^/V were 0.35 and 0.485, respectively, while for SDF, the values were 5.02 and 6.99, respectively. For SDGM, (O-E)^2^/E and (O-E)^2^/V were 9.53 and 12.12, respectively, while for SDGF, the values were 1.16 and 1.54, respectively].

Additional pairwise analyses were performed to determine the nature of the difference. The survival of male and female mice provided with the standard diet over a period of 912 days is illustrated in Figure 3B. Comparison of the Kaplan–Meier plots for SDM (observed events—59) and SDF (observed events—41) indicated *p =* 0.370 (log-rank test). [For SDM, (O-E)^2^/E and (O-E)^2^/V were 0.34 and 0.80, respectively, while for SDF, the values were 0.42 and 0.80, respectively].

The survival of male and female mice provided with the grape-supplemented diet over a period of 912 days is illustrated in Figure 3C. Comparison of the Kaplan–Meier plots for SDGM (observed events—64) and SDGF (observed events—45) indicated a statistically significant increase in the survival of female mice (*p* = 0.009, log-rank test). [For SDGM, (O-E)^2^/E and (O-E)^2^/V were 3.34 and 6.89, respectively, while for SDGF, the values were 2.93 and 6.89, respectively].

However, when the male or female groups consuming the standard diet versus the standard diet supplemented with grapes were compared, no statistically significant differences were noted. The Kaplan–Meier plots shown in Figure 3D, comparing SDM (observed events—59) and SDGM (observed events—64), yielded *p* = 0.185 (log-rank test). [For SDM (O-E)^2^/E and (O-E)^2^/V were 0.77 and 1.75, respectively, while for SDGM, the values were 0.905 and 1.75, respectively].

Finally, the Kaplan–Meier plots shown in Figure 3E, comparing SDF (observed events—41) and SDGF (observed events—45) yielded *p* = 0.511 (log-rank test). [For SDF, (O-E)^2^/E and (O-E)^2^/V were 0.22 and 0.43, respectively, while for SDGF, the values were 0.18 and 0.43, respectively].

### 2.4. Effect of Grapes on Gene Expression in the Kidney

#### 2.4.1. Venn Diagrams

**Figure 4:**
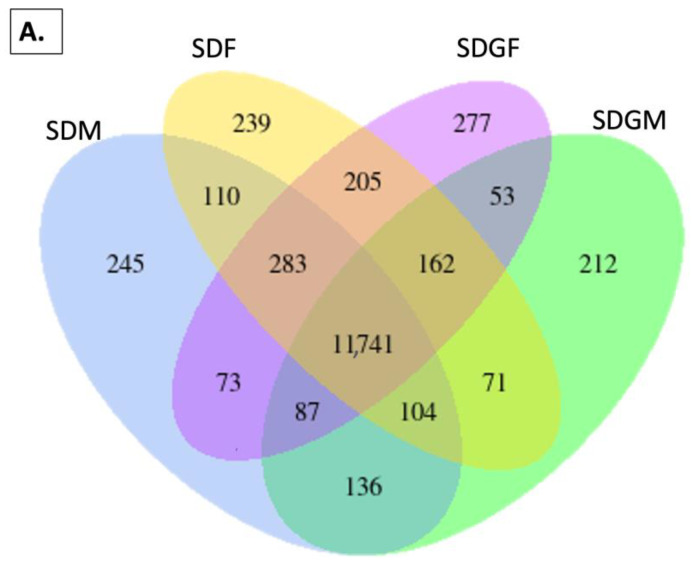

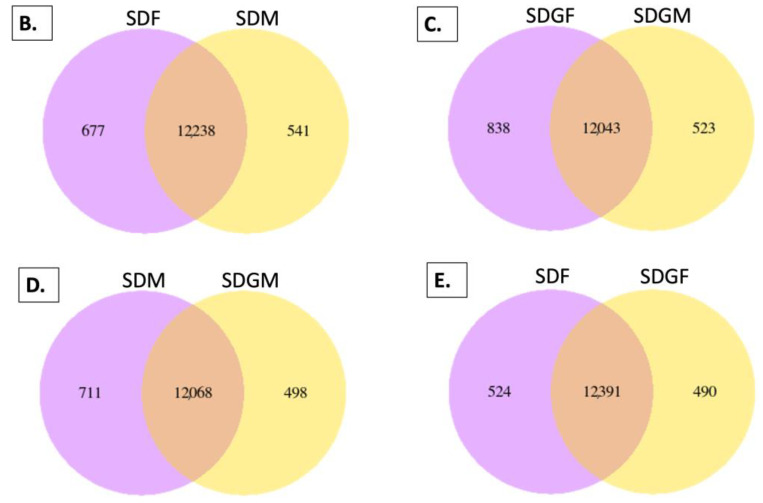
Venn diagrams depicting genes expressed in the kidney of mice provided with standard and grape-supplemented diets. (**A**) Venn diagram of genes expressed by the four groups of mice illustrating genes co-expressed (overlapping regions) and uniquely expressed (non-overlapping regions) among all groups. The default threshold of the FPKM value is set to 1 for the selection of the genes for each group. (**B**) Venn diagram of genes expressed by SDF and SDM. (**C**) Venn diagram of genes expressed by SDM and SDGM. (**D**) Venn diagram of genes expressed by SDGF and SDGM. (**E**) Venn diagram of genes expressed by SDF and SDGF.

Although lifespan was not extended under these experimental conditions, based on the significant differences in the kidney/body weight ratios induced by the dietary grape supplementation, we investigated the kidney in greater detail. The approach used was an RNA-seq analysis of the kidney tissue and an investigation of differential expression patterns across genders and dietary treatments. Figure 4 illustrated the distribution of these genes by Venn diagrams, highlighting both shared and unique gene subsets among the experimental groups. As shown in Figure 4A, comparison of all four groups demonstrated that the majority of genes were expressed in common. Importantly, however, approximately 200 genes were uniquely expressed by each of the four groups, and various patterns of shared expression were observed. Pairwise comparisons of males and females provided with the standard diet (Figure 4B) or the standard diet supplemented with grapes (Figure 4C) revealed unique expression patterns due to gender, and direct pairwise comparisons of the respective genders also revealed unique expression patterns resulting from dietary grapes in both males (Figure 4D) and females (Figure 4E).

#### 2.4.2. Principal Component Analyses

**Figure 5:**
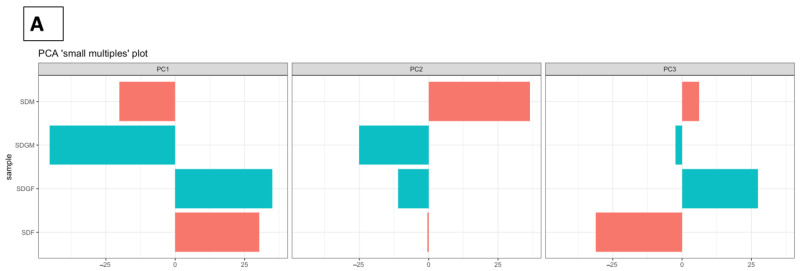

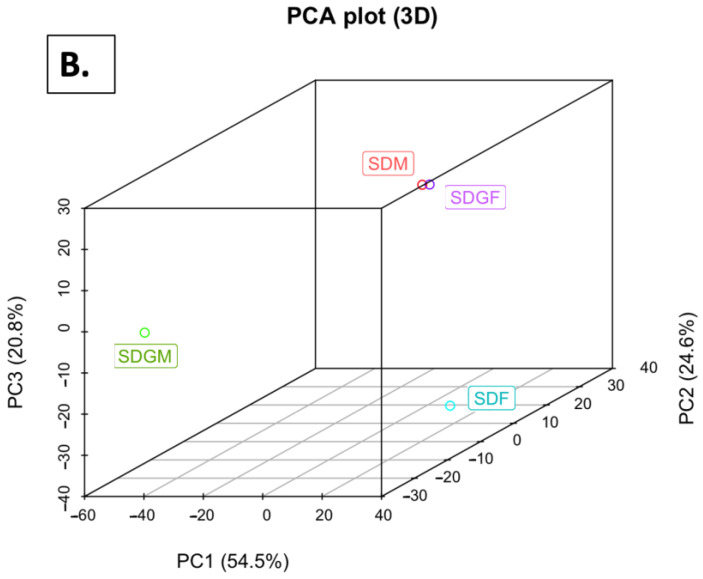
Principal component analysis (PCA) plots for kidney specimens influenced by gender and diet. (**A**) The bars represent the variance contributed by each principal component (PC1, PC2, and PC3). Longer bars indicate PCs that capture more variability in the data. (**B**) PCA plot presented in 3D visualization.

As a first attempt to visualize the patterns of gene expression variation, PCA analysis was conducted (Figure 5). PC1 demonstrated separation primarily along gender lines, indicating gender-specific clustering patterns. However, a significant divergence, suggesting distinctions in gene expression profiles influenced by dietary regimens, was observed in males (PC2) and females (PC3) (Figure 5A). These combined observations of differences in variance are illustrated in a 3D PCA plot, where SDM and SDGF map closely but distal from their respective dietary counterpart (Figure 5B). Overall, the results signify fundamental differences based on gender, as well as less profound yet meaningful differences within each gender due to the addition of grapes to the diet.

#### 2.4.3. Volcano Plots

**Figure 6:**
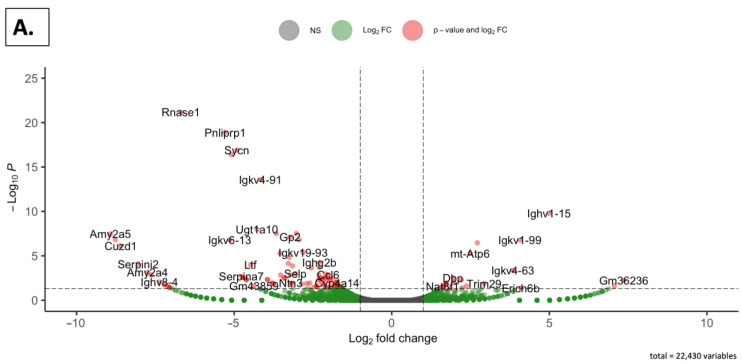

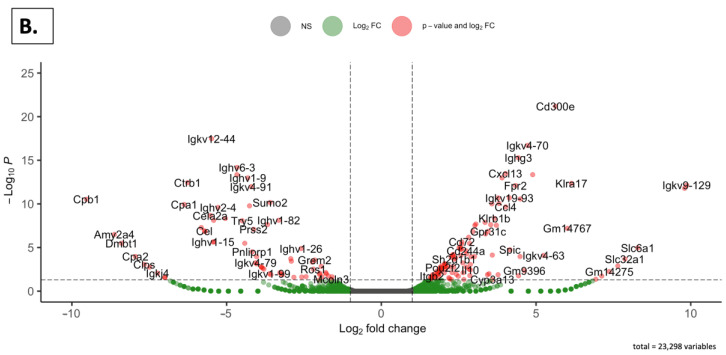
Alteration of gene expression in mouse kidney by dietary grapes. (**A**) Volcano plot for SDGM vs. SDM showing up-regulated, down-regulated, and unaltered genes (dots) in the kidney. (**B**) Volcano plot for SDGF vs. SDF showing up-regulated, down-regulated, and unaltered genes (dots) in the kidney. In each case, the threshold for differentially expressed genes was set as |log_2_(fold-change)| > 1 and −log_10_(Padj) > 1.3 (Padj < 0.05). Gene names are designated next to the dots.

To further evaluate the gene expression alteration induced by the dietary intervention in the two genders, volcano plots were generated. Significance thresholds were set to identify differentially expressed genes (DEGs). As shown in Figure 6A, when comparing the SDM and SDMG groups in a plot that included 22,430 genes, 21 genes were up-regulated, and 108 genes were down-regulated. For the SDF and SDGF groups (Figure 6B), in a volcano plot that included 23,298 genes, 109 genes were up-regulated, and 65 genes were down-regulated.

#### 2.4.4. Heat Maps

**Figure 7:**
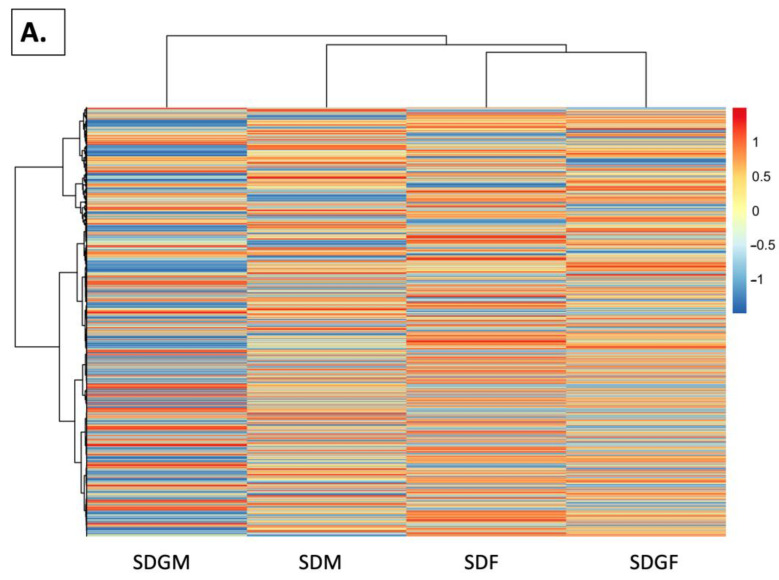

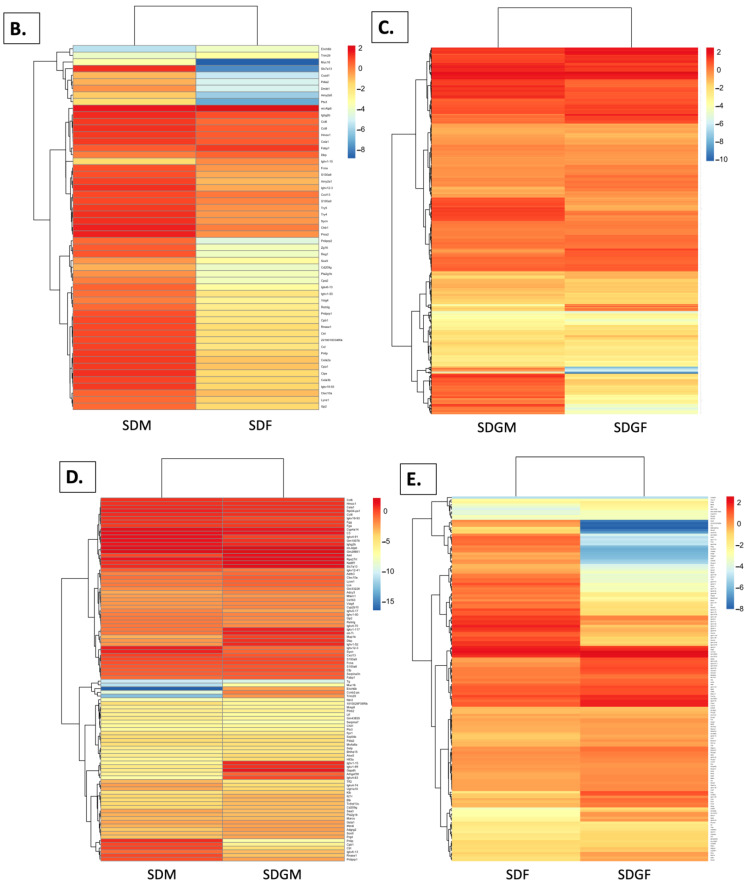
Heat maps illustrating gene expression in the kidneys of male and female mice provided with standard diet and standard diet supplemented with grapes. (**A**) Heatmap showing expression changes (*z*-score) for global genes in the kidney. Heatmap showing the expression of the DEG list for the comparison of (**B**) SDM vs. SDF, (**C**) SDGF vs. SDGM, (**D**) SDGM vs. SDM, and (**E**) SDGF vs. SDF.

Next, to visualize the global expression patterns and clustering of all genes, Ward’s linkage method was employed, and dendrograms were constructed. The expression profiles of the clusters provided patterns for both the gender and treatment effects. As shown in Figure 7A, the female mice with the standard diet mapped most closely with the female mice with the grape diet, as was the case with the male mice. As might be anticipated from the PCA analysis, the greatest variation was noted with the males provided with the grape diet versus the females provided the grape diet.

Visualization of DEGs through heatmap analysis was also applied to visualize the comparisons of gene expression patterns across the groups. Expression values were normalized using *z*-score transformation, enabling the contrast and visualization of differences in gene expression patterns. As shown in Figure 7B, the comparison of male and female mice provided with the standard diet revealed major differences in the homeostatic state due to gender. Interestingly, when grapes were added to the diet, the comparative expression patterns between the genders demonstrated greater similarity (Figure 7C). As expected, to yield such a comparative change, expression patterns were modulated when comparing the male groups provided with the standard or grape-supplemented diets (Figure 7D) and with the female groups provided with standard or grape supplemented diets (Figure 7E). The variation appeared to be greater in females than in males.

#### 2.4.5. FPKM Value Plots

**Figure 8:**
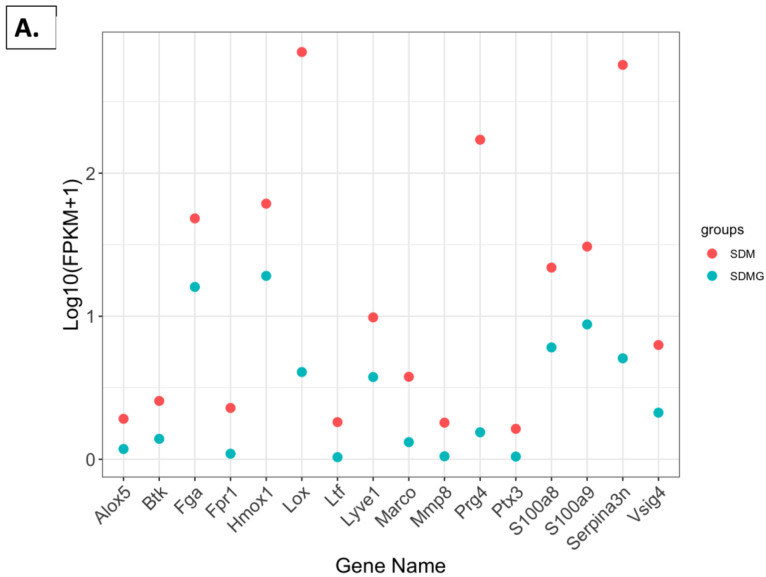

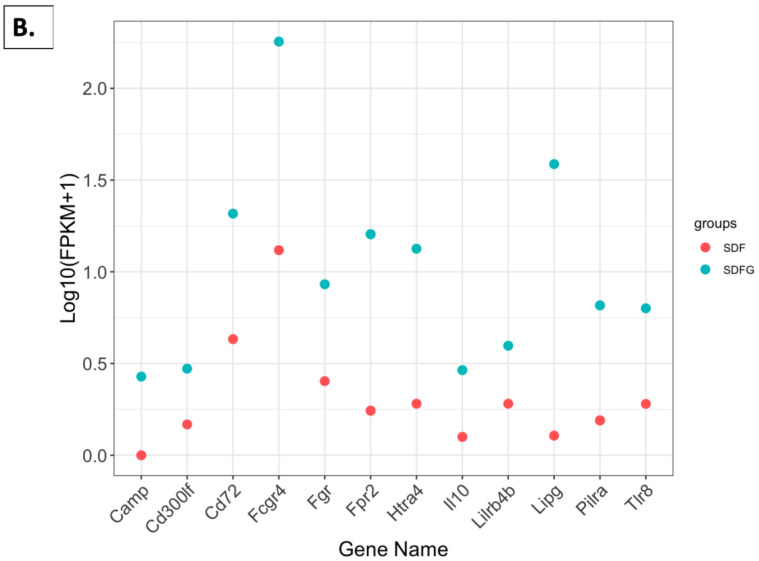
Genes affected by grapes in males and females. (**A**) SDMG vs. SDM based on the log10(Fpkm + 1) values of the indicated genes. (**B**) SDFG vs. SDF based on the log10(Fpkm + 1) values of the indicated genes.

Normalized expression levels of males (Figure 8A) and females (Figure 8B) were examined by fragments per kilobase of transcript per million mapped read (FPKM) values. These genes were selected from enriched pathways and further filtered to meet the criterion of having a padj value less than or equal to 0.05. In males provided with the grape diet, genes including *alox5*, *btk*, *fga*, *fpr1*, *hmox1*, *lox*, *ltf*, *lyve1*, *marco*, *mmp8*, *prg4*, *s100a8/9*, *serpina3n*, and *vsig4* showed lower log10(fpkm + 1) values compared to the standard diet group. Conversely, in females provided with the grape diet, genes including *camp*, *cd300lf*, *cd72*, *fcgr4*, *fgr*, *fpr2*, *htra4*, *il10*, *lilrb4b*, *lipg*, *pilra*, and *tlr8* showed higher log10(fpkm + 1) values compared to the standard diet group.

#### 2.4.6. FPKM Values Related to Immune Function

In addition to the direct effects on the tissue function, it was speculated that grape consumption may alter the immune function. Therefore, we specifically investigated proinflammatory genes consisting of chemokines, interleukins, cytokines, and immune receptors for the comparison of SDGM vs. SDM in the kidney. Comprehensive lists derived from the kidney are presented in Supplemental Tables S3 and S4. Similarly, comprehensive lists of the immune-related genes derived from the liver are shown in Tables S5 and S6. Most genes showed no significant difference when comparing mice provided with the standard versus grape-supplemented diet. However, as shown in Table 3 with the kidney, up-regulated genes in the SDGM group included *cxcl13*, *ccl4*, *ccl3*, and *il10*, and down-regulated genes in the SDGF group included *cxcl13*, *ccl6*, *ccl8*, and *ccl24*. In the SDGM group, a larger group of proinflammatory genes were altered. Similar to the kidney, *Cxcl13* and *Ccl6* were down-regulated, as well as *Il1b*, *Cxcl14*, *Lepr, Pglyrp1*, *Tnfsf14*, and *Il10ra*. On the other hand, U6 and 7SK were up-regulated (Table S5). In the SDGF group, similar to the kidney, *Cxcl13* and *Ccl4* were up-regulated in the liver, as well as *Cxcl14*, *Ccl4,* and *Ccr5*. *Lepr* was down-regulated (Table S6).

### 2.5. Reactome Pathway, KEGG Pathway, and GSEA Analysis with Kidney

**Figure 9:**
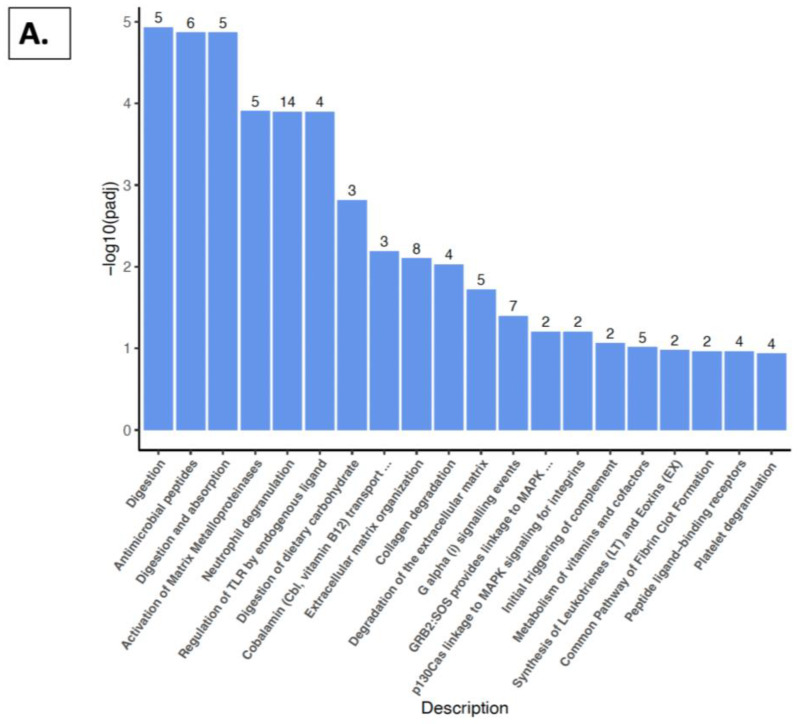

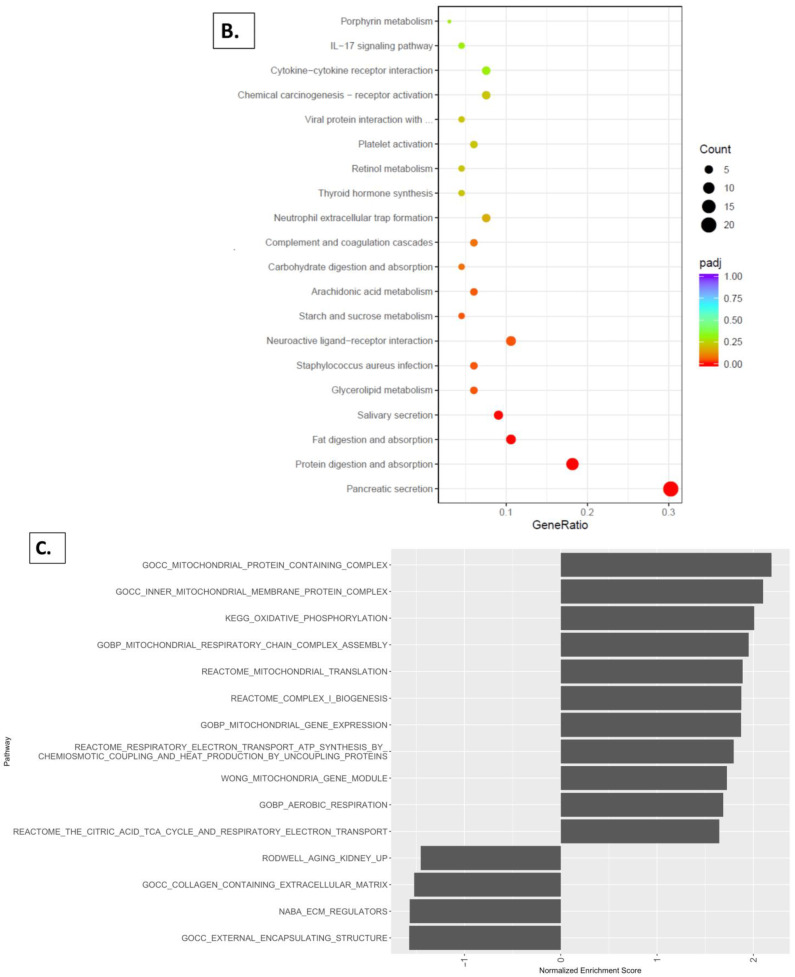
Pathway analysis comparing SDGM vs. SDM groups. (**A**) Reactome pathway analysis. (**B**) KEGG analysis. (**C**) GSEA showing normalized enrichment scores.

**Figure 10:**
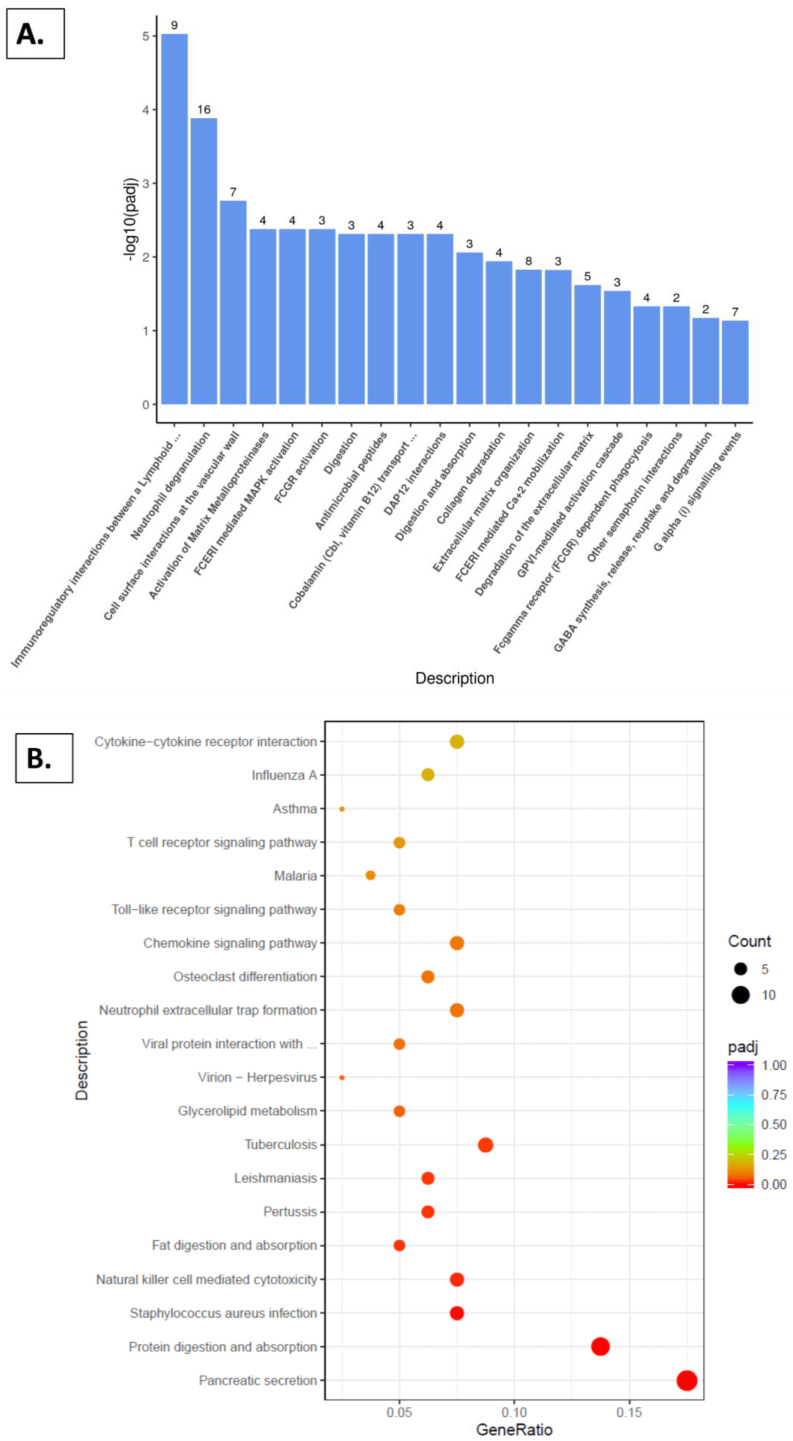

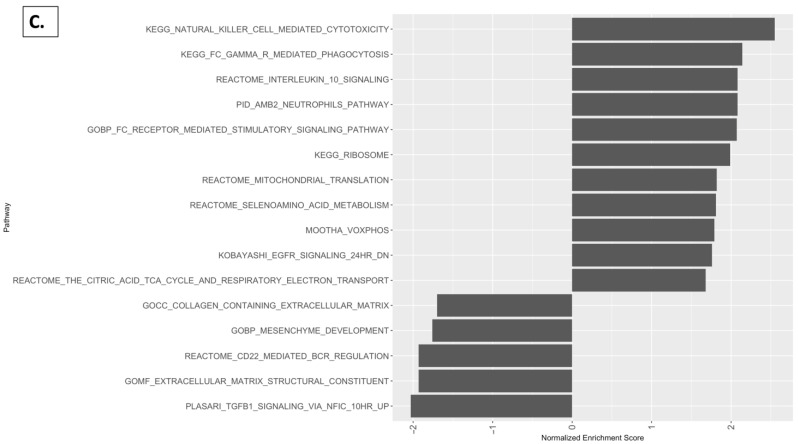
Pathway analysis comparing SDGF vs. SDF groups. (**A**) Reactome pathway analysis. (**B**) KEGG analysis. (**C**) GSEA showing normalized enrichment scores.

The molecular pathways underlying these variations in gene expression patterns, based on diet and/or gender, were then examined. In both the males and females, pathway analysis showed significant alterations associated with several physiological functions. Notably, an enrichment was observed in pathways related to immune function, collagenase degradation, extracellular matrix regulation, metabolism of vitamins and cofactors, pancreatic secretion, aging, and mitochondrial function (Figure 9 and Figure 10).

Figure 9A,B illustrate the Reactome and KEGG pathways, respectively, for male mice. Comparison between the groups provided with the grape and standard diets showed an enrichment in several pathways, including collagenase degradation, antimicrobial peptides, cobalamin transport and metabolism, protein digestion and absorption, neutrophil degranulation, and regulation of the Toll-Like Receptor (TLR). Additionally, Gene Set Enrichment Analysis (GSEA) (Figure 9C) revealed an enrichment in the pathways associated with mitochondrial translation, electron transport, and ATP synthesis, accompanied by a reduction in the pathways linked to aging, collagenase extracellular matrix (ECM), and ECM regulators.

Analyses of the Reactome and KEGG pathways (Figure 10A,B), comparing the female groups provided with the grape diet and standard diet, demonstrated an enrichment of the pathways similar to those observed with the male groups (Figure 9A,B). Specifically, an enrichment of the immunoregulatory interactions, neutrophil degranulation, antimicrobial peptides, glycerolipid metabolism, and pancreatic secretion was observed. The GSEA analysis (Figure 10C) revealed an enrichment in the pathways including IL-10 signaling, as well as the neutrophil pathway and Fc-gamma receptor (FcγR) pathway. Additionally, the pathways related to the tricarboxylic acid (TCA) cycle, respiratory electron transport, mitochondrial translation, and amino acid metabolism showed an enrichment. Conversely, a negative enrichment was observed in the pathways related to the ECM structural constituents, collagen-containing extracellular matrix, CD22-mediated BCR regulation, and TGFB1 signaling.

## 3. Discussion

Our main object in this work was to assess how control animals given what may be termed a “bland” diet might differ from animals given the same diet supplemented with grapes. This was accomplished with a mouse model, using diets that were essentially matched, other than including the grape phytochemical components in the supplemented diet. Based on relative body weight, daily food consumption, and standard mammalian conversion factors [15], the addition of 5% grape powder was selected since this roughly equates to the human consumption of two servings of grapes per day [a ¾ cup serving of fresh grapes (126 g) is equivalent to 24 g of the commission freeze-dried whole table grape powder] [16]. Both male and female mice were included in the study to assess the potential of variation due to gender.

As illustrated in Figure 1, the body weight of male mice was greater than the body weight of females through the course of the study. However, with the respective groups, there was no difference when comparing the standard diet versus the standard diet supplements with grape powder. Thus, it is clear that the diets were designed to be sufficiently matched.

In the previous work, under dietary duress, we have shown some beneficial effects when comparing female mice given a high-fat diet versus female mice given a high-fat diet supplemented with grapes [13], including enhanced longevity. Naturally, we were interested to learn if any effect on longevity was mediated by grape supplementation in the absence of a high-fat diet, and if any differences exist based on gender. It was noted that the survival of the females provided with the grape diet was longer than the males provided with the grape diet (*p* = 0.009). The gender advantage of the female mice in terms of longevity has been noted previously [17,18]. For example, with the female C57BL/6J mice, the mean longevity was 561 days, whereas for males, the mean longevity was 539 days.

However, in our study, a comparison of males and females, when both groups were provided with the standard diet, revealed no significance difference in longevity (*p* = 0.370). Further, a direct comparison of each gender provided with a standard diet vs. a diet supplemented with grapes did not reveal any significant differences (*p* = 0.185 and 0.511 for males and females, respectively). Given the positive trend with male mice (*p* = 0.185), it may be that statistical significance could be achieved under different experimental conditions or with a larger group size.

In addition to overall survival, we considered the potential effect of the grape intake on percentage of survival along the way to the end of the study. Given the isocaloric properties of the diets, it is assumed that body weight correlates with intake. In regard to the survival rate, the data are presented in Supplemental Tables S1 and S2, for males and females, respectively. At the end of the study, i.e., no surviving mice, there was no significant difference between the respective groups. With female mice, there were no significant differences at 75, 50, 25, or 0% survival. However, with males, a difference was discerned at 75% survival, which was reversed at 50% survival and showed no difference at 25 or 0% survival. Given these patterns, it seems clear that intake does not have a bearing on female survival. The shifting pattern with males is difficult to interpret, but it does not seem reasonable to attribute the results to grape consumption per se.

In retrospect, this generalized lack of effect is not surprising. It should be remembered that the mice employed for the study were a normal, healthy inbred species. They were provided with a high-quality semi-synthetic pure diet, HEPA-filtered air, perfectly controlled temperature and humidity, frequent cage changes, environmental enrichment, and constant medical care. Under these ideal environmental conditions, shielded from any real-world stress, it is difficult to envision any reason that lifespan would not be optimal from the outset. Although it is possible that long-term grape consumption could yield an effect with free-living animals, this is challenging to assess.

Unlike life span, health span is a more difficult parameter to evaluate. The mice subjected to more detailed analysis in this study were 2.5 years of age, which may be roughly correlated to 80 years of age for a human being [19]. At this point in time, approximately 60% of the mice originally entered into the study had expired, and significant decreases in body weight were being observed in all groups, indicative of decline.

As a generic assessment of their state of health and the following recommended practice for toxicology studies [20], body-weight-to-organ-weight ratios were determined. No significant differences were observed with the liver, colon, muscle, prostate, or ovary (Table 1). In addition, a histopathological evaluation of all the designated tissues did not reveal any profound differences between the groups. However, with the kidney, relative to the groups provided with the standard diet, the groups provided with the grape supplemented diet (male and female), the weights were significantly lower than the control groups.

As a rough comparison, Jackson Labs reports the kidney to be equivalent to 0.51 ± 0.08% body weight for female C57BL/6J mice (26 weeks) and 0.58 ± 0.06% body weight for males (78 weeks) [21]. In the current study, with the standard diet, the kidney corresponded to 0.98 and 0.69% for males and females, respectively, and this was reduced to 0.70 and 0.59%, respectively, when the grape was added to the diet. Given the age of the mice in our study was 130 weeks, we interpret this as trending toward a normal younger mouse.

As with the liver, colon, muscle, prostate, or ovary, the histological evaluation of the kidney did not reveal profound differences between the groups. Overall, male and female mice given the standard diet predominantly showed chronic interstitial inflammation, compared to mice given the grape-supplemented diet, showing perivascular inflammation. The blood chemistry between the groups was also not profoundly different. However, the BUN levels tended to be higher in the male and female groups given the standard diet relative to the grape diet, although statistical significance was not achieved. Nonetheless, it is notable that the average values when provided with the grape-supplemented diet, for both males and females, fell within the reference values, whereas the average values when given the standard diet were elevated. Creatinine (CRE) values all fell within the reference range. Considering the BUN-to-creatinine ratios, based on the average reference values, the ratio was 46.7 for both males and females. With the mice provided with the standard diet, based on the averages, the ratios were 125 and 152 for males and females, respectively, and reduced to 63.7 and 40.4, respectively, when provided with the grape diet. There is a large body of literature regarding high BUN:CRE ratios, which may be indicative of a variety of maladies, including acute renal failure, chronic kidney disease, glomerulonephritis, dehydration, and heart failure. In decompensated patients with heart failure, an elevated BUN/creatinine is strongly associated with death [22]. In sum, although partially based on conjecture, we decided to explore any fundamental differences between the kidneys of the aged mice provided with the standard diet versus the aged mice provided with a long-term administration of dietary grapes. The approach we used was based on the analysis of phenotypic expression.

On a macrolevel, considering the unique expression of over 200 genes (Figure 4), it is clear that the male mice can be differentiated from the female mice, and, moreover, well-nurtured animals given dietary grapes were clearly differentiated from well-nurtured animals given a matched diet devoid of grapes. This is consistent with the PCA (Figure 5) and volcano (Figure 6) plots. Comparisons of DEG lists utilizing heatmaps again illustrated differences between genders (Figure 7B) as well as differences induced by the addition of grapes to the diets with both genders (Figure 7D,E), but the visual inspection of the heatmaps of both genders provided with the grape-supplemented diet (Figure 7C) suggests movement to a state of greater similarity. The comparative analysis of FPKM values supports this process of normalization, wherein gene expression in the males is regulated downward (Figure 8A) and gene regulation in the females is regulated upward (Figure 8B). It should be noted that the genes listed and quantified for the males in Figure 8A are also expressed in the females, and the genes listed and quantified for the females in Figure 8B are also expressed in the males, but there are no statistically significant shifts induced by grape consumption (Supplemental Tables S7 and S8).

Moreover, in each case, the gene shifts illustrated in Figure 8 may be of relevance to the kidney function. For example, Alox5 mediates acute injury and has been implicated in interstitial fibrosis and chronic kidney disease (CKD) progression [23]. BTK interacts with p53 and MDM2, enhancing p53 activity; its inhibition may reduce senescent cells in tissues, potentially impacting aging [24]. The aggregation of the fibrinogen Aα chain (FGA) is linked to renal amyloidosis [25]. Formyl peptide receptor 1 (FPR1) is abundant in neutrophils and regulates intracellular calcium, which plays a crucial role in diabetic nephropathy [26]. *Hmox1* induction helps mitigate oxidative stress-mediated damage and inflammation, contributing to improved kidney function in diabetic kidney disease [27]. *LOX* inhibition reduces renal fibrosis by decreasing collagen deposition, presenting *LOX* as a therapeutic target for renal fibrosis [28]. *LTF* expression increases with age and is implicated in neurodegenerative diseases [29]. *Lyve1* marks lymphatic endothelial cells and plays a significant role during inflammation, which is critical in acute kidney injury (AKI) and its transition to CKD [30]. The MARCO receptor is crucial for the phagocytosis of poly-methyl methacrylate particles by aged mouse peritoneal macrophages, leading to increased pro-inflammatory cytokine production [31]. *S100A8/A9* mediates renal damage and fibrosis through the loss of tubular epithelial cell contacts [32]. *SerpinA3c/k* relocates to the apical tubular membrane in CKD, suggesting secretion in pathological contexts [33]. *VSIG4* is involved in renal aging and may contribute to age-related kidney alterations [34]. The reduction in FPKM values for each of these factors in male mice suggests a beneficial effect of grapes.

Conversely, elevated FPKM values in female mice are suggestive of beneficial kidney effects. For example, emerging evidence indicates that immune receptors are involved in aging-related processes such as metabolism, inflammation, and cognitive decline. *CD300f*, a *TREM2*-like receptor, integrates cell-signaling pathways to modulate inflammation and microglial fitness, contributing to healthy aging [35]. *FPR2* exerts anti-inflammatory effects and suppresses disease progression in various organs [36]. *Htra4* plays a key role in protein quality control by managing protein folding stress and regulating signal transduction [37]. Il10 helps to maintain tissue homeostasis during severe infection in kidney disease by inhibiting excessive inflammatory responses and promoting tissue repair [38]. *Lilrb4* is an inhibitory receptor involved in immune checkpoint pathways, regulating multiple immune responses [39].

Another aspect of grape action that warrants particular attention is the potential to mediate immunomodulation. Many well-known proinflammatory cytokines (e.g., IL-1, IL-6, and TNF-α) were not altered by grape consumption (Tables S3 and S4). However, as summarized in Table 3, we found that *cxcl13*, *ccl4*, *ccl3,* and *il10* were up-regulated in females while *cxcl13*, *ccl6*, *ccl8,* and *ccl24* were down-regulated in males.

The up-regulation of *cxcl13* in females suggests increased B-cell infiltration, as cxcl13 is known to promote this process [40]. Additionally, *ccl3* and *ccl4*, which are responsible for the recruitment of inflammatory cells [41], were increased in females. The up-regulation of *il10*, a specific anti-inflammatory cytokine, indicates a mechanism to inhibit excessive inflammatory responses, potentially balancing the inflammatory environment [38].

In contrast, males exhibited a down-regulation of *ccl6*, a chemokine primarily produced in monocytes [42], and *ccl8*, which is associated with kidney fibrosis [43]. The down-regulation of *ccl24*, a pro-fibrotic and pro-inflammatory chemokine expressed in fibrotic diseases, further supports a reduction in fibrotic and inflammatory activity in males [44]. These differential cytokine expression patterns, as well as those observed in the liver (Tables S5 and S6), suggest complex immunomodulatory effects mediated by grape consumption that are gender specific.

Finally, as an attempt to gain a more comprehensive understanding of the influence of grape consumption on kidney function, a comparative analysis of SDGM vs. SDM and SDGF vs. SDF was performed using the Reactome pathway analysis, KEGG analysis, and GSEA normalized enrichment scores.

Several pathways related to immune function, collagenase degradation, extracellular matrix (ECM) regulation, metabolism of vitamins and cofactors, pancreatic secretion, aging, and mitochondrial function were enriched in both males and females (Figure 9 and Figure 10). These findings demonstrate that a grape diet significantly enhanced the mitochondrial function in the kidney. This is crucial for maintaining various cellular processes, including the regulation of reactive oxygen species (ROS), cytosolic calcium levels, and apoptosis, as well as ATP production, which is vital for basal cell functions, cellular repair, and regeneration [45]. Potentially, tubules can be exposed to bioactive molecules originating from the plasma or inflamed glomeruli and appear in the glomerular ultrafiltrate [46]. For example, transferrin protein present in tubular fluid, provides a ready source of ferric iron that can catalyze the Haber-Weiss–Fenton reaction, generating damaging ROS. Transferrin-bound iron has toxic effects on cultured tubular cells [47]. The regulation of ROS can adapt to different metabolic conditions through several signaling pathways such as mechanistic target of rapamycin (mTOR) and AMP-activated protein kinase (AMPK). These pathways activate the transcriptional co-activator peroxisome proliferator-activated receptor-γ co-activator 1α (PGC1α) and balance mitochondrial dynamics and energetics to maintain mitochondrial homeostasis. This recognition emphasizes the requirement for cellular homeostasis and the potential vulnerabilities that can arise from dysregulated bioactive molecules and iron metabolism within the tubular environment. Further studies would be of interest to help elucidate the mechanisms capable of mitigating the adverse effects of ROS.

Additionally, our results indicate that the grape diet impacts the renal extracellular matrix (ECM). The ECM, comprising collagens, elastin, glycoproteins, and proteoglycans, undergoes significant remodeling during fibrosis and plays a significant role in inflammation [48]. Additionally, mutations in proteoglycan genes have been linked to many genetic diseases [49,50]. Glycoproteins (characterized by a group of proteins with covalently attached oligosaccharide chains) and proteoglycans (glycosaminoglycans covalently bound to a protein core) modulate signaling pathways. For example, small leucine-rich proteoglycans (SLRPs) activate the epidermal growth factor receptor (EGFR), insulin-like growth factor 1 receptor (IGFIR), and low-density lipoprotein-receptor-related protein 1 (LRP1). SLRPs also regulate inflammatory responses by binding to and activating TGF-β [51,52].

The negative enrichment of collagen expression is also notable, in that collagens typically accumulate in the renal interstitium during fibrosis [53]. For example, post kidney injury, it has been shown that an increase in profibrotic cytokine activity within the inflamed microenvironment leads to the activation of matrix-producing cells, a central event in renal fibrogenesis [54,55]. Various types of cells in the tubulointerstitium of the kidneys can produce ECM, such as fibroblasts, tubular epithelial cells, vascular smooth muscle cells, and a subset of macrophages. However, fibroblasts are commonly regarded as the principal matrix-producing cells that generate a large amount of interstitial matrix components, including fibronectin and type I and type III collagens [56,57]. The activation and proliferation of these cells contribute significantly to the excessive deposition of ECM.

In males, the Reactome and KEGG pathway analyses (Figure 9A,B) showed an enrichment in pathways such as collagenase degradation, antimicrobial peptides, cobalamin transport and metabolism, protein digestion and absorption, and neutrophil degranulation, as well as the regulation of TLR signaling. The GSEA (Figure 9C) highlighted the pathways associated with mitochondrial translation, electron transport, and ATP synthesis. Conversely, there was a reduction in pathways linked to aging, ECM, and ECM regulators.

For females, the Reactome and KEGG pathway analyses (Figure 10A,B) indicated an enrichment in immunoregulatory interactions, neutrophil degranulation, antimicrobial peptides, glycerolipid metabolism, and pancreatic secretion. The GSEA (Figure 10C) showed enrichment in IL-10 signaling, neutrophil pathways, and Fc-gamma receptor (FcγRs) pathways. IL-10 signaling was positively enriched with the grape diet. IL-10 inhibits the expression of the fibrosis-related miRNAs induced by transforming growth factor-β (TGF-β), playing a crucial role in fibrosis prevention [58]. The inhibition of IL-10 is linked to increased renal interstitial fibrosis, severe renal tubular injury, collagen deposition, and a higher expression of pro-fibrotic genes [59]. Furthermore, the pathways related to the tricarboxylic acid (TCA) cycle, respiratory electron transport, mitochondrial translation, and amino acid metabolism were enriched, while pathways associated with ECM structural constituents, collagen-containing ECM, CD22-mediated BCR regulation, and TGFB1 signaling were negatively enriched. The grape diet negatively influenced the TGF-β1 expression, which is associated with severe glomerulonephritis and glomerulosclerosis [60].

In sum, using a murine model and a nutrigenomic approach, we have demonstrated the ability of dietary grapes to significantly modulate the expression of a vast array of genes differentially in males and females. Yet, with both genders, a specific effect on the kidney was observed. We have attempted to interpret our results in a manner that is consistent with the end result, realizing at the same time that many other factors may come into play. Of note, grape consumption affects the microbiome [9], and the gut kidney axis [61] may have some bearing on our results. Similarly, metabolomic alterations [14] may come into play. Realistically, from a holistic viewpoint, a myriad of factors, well beyond what we have been able to decipher, are likely to influence outcome. Nonetheless, the findings reported herein suggest that a grape diet positively modulates mitochondrial function and various immune pathways while negatively impacting fibrosis-related pathways in the kidney. This modulation could potentially contribute to renal health and resilience against fibrosis and related pathologies.

## 4. Materials and Methods

### 4.1. Animals and Diets

#### 4.1.1. Semi-Synthetic Diets

To ensure consistency and continuity in experimental and clinical research on the biological and physiological potential of grapes, a freeze-dried powder is produced under the supervision of the California Table Grape Commission (Fresno, CA, USA). This grape powder, which acts as a substitute for fresh grapes, is made from fresh seeded and seedless red, green, and black grapes that are ground and freeze-dried to preserve their bioactive compounds. For the current studies, standardized freeze-dried grape powder, prepared and analyzed as previously described [62], was provided in vacuum-sealed packets and stored at −20 °C to maintain the stability of its phytochemical components. To further ensure quality, the standard product underwent microbial analysis and was found to be contaminant-free [62].

Based on the composition of grapes, paired isocaloric diets were custom-designed and produced by Envigo (Madison, WI, USA) as follows: a 4% fat standard diet (SD; TD.160157) and a standard diet plus 5% (*w*/*w*) standardized grape powder (SDG; TD.160158) (Table 4). As previously described, adding grape powder in this manner to these diets does not significantly affect the rate of consumption [63].

#### 4.1.2. Animal Protocol

A total of 240 male and 240 female C57BL/6J mice, obtained from The Jackson Laboratory (Bar Harbor, ME, USA) at 4 weeks of age, were provided with a standard diet (SD). At 1 year of age, both sexes were randomly assigned to four equal groups (120 per group). Half of the mice continued on the SD diet, while the other half were switched to a standard diet containing 5% grape powder (SDG). The group sizes were determined based on a power analysis conducted using a G*Power a priori test (effect size calculated at 0.3; alpha error probability at 0.05; degrees of freedom at 1; version 3.1.9.7). The mice were housed in ventilated racks with HEPA-filtered air delivered to each cage (4 animals per cage). The temperature (21 ± 2 °C) and humidity (30–70%) were carefully controlled, with a 12 h light–dark cycle. No other animals were housed in the same room, except for the sentinel animals used for the health monitoring program. Food and water were provided ad libitum throughout the lifetime of the mice. Each mouse was implanted with a radio-frequency identification (RFID) microchip (Unified Information Devices Inc., Lake Villa, IL, USA) for permanent identification. Body weight was measured biweekly throughout the study.

In each group, 100 mice were monitored for survival, while 20 mice were maintained under identical conditions and held in reserve. If a mouse in any group of 100 needed to be removed due to abnormal circumstances (such as a wound), it was replaced with a randomly selected mouse from the respective group of 20. This ensured that only mice dying of natural causes were included in the survival analyses.

This study was conducted in accordance with an Institutional Animal Care and Use Committee (IACUC) protocol approved by Baystate Health, Springfield, MA, USA (protocol number 1736198-2 approved 20 April 2021).

#### 4.1.3. Tissue and Serum Collection

Five mice from each of the four groups, aged 2.5 years, were randomly selected and euthanized after an overnight fasting period. Body weights were recorded. Euthanasia was performed using CO_2_ followed by cervical dislocation. Blood was collected via cardiac puncture in lithium-heparin tubes and centrifuged at 3000× *g* for 10 min to prepare plasma.

The liver, colon, kidney, muscle, ovary or prostate were harvested and weighed. The tissues were immediately submerged in RNAlater™ stabilization solution (ThermoFisher Scientific, Waltham, MA, USA) or fixed in 10% neutral buffered formalin (NBF). RNAlater specimens were stored at room temperature for up to 12 h and then transferred to −20 °C until RNA extraction.

For the kidney and ovary, each entire organ was placed in either RNAlater or NBF. The colon was measured and then halved, with each half placed in RNAlater or NBF. For the liver, a small portion of both the left and right lobes was placed in RNAlater, another part was placed in NBF, and the remainder was frozen. The thigh muscle and prostate were halved, with each half placed in either RNAlater or NBF.

### 4.2. Blood Analysis

Approximately 100 µL of whole blood was collected in lithium-heparin tubes for blood chemistry testing. The “Comprehensive Diagnostic Profile” in the VetScan VS2 analyzer (Abaxis) was selected to measure alanine aminotransferase (ALT), albumin (ALB), alkaline phosphatase (ALP), amylase (AMY), calcium (Ca), creatinine (CRE), globulin (GLOB), glucose (Glu), phosphorus (PHOS), potassium (K), sodium (Na), total bilirubin (TBIL), total protein (TP), and blood urea nitrogen (BUN). The remainder of the blood was centrifuged, and the plasma was stored frozen.

### 4.3. Histopathological Examination of Kidney and Other Tissues

One kidney from each mouse was fixed in 10% NBF and then transferred to 70% ethanol. The samples were dehydrated in a graded series of ethanol solutions (70%, 95%, and absolute ethanol), cleared in xylene, and infiltrated with paraffin at 60 °C using the Leica ASP300S processor. The tissues were subsequently embedded in paraffin molds using the Leica EG1150C. For histopathological evaluation, serial sections were cut at a thickness of 4 µm, deparaffinized in xylene and rehydrated through graded ethanol (100% and 90%), stained with hematoxylin and eosin (H&E) or Masson’s trichrome, dehydrated again in graded ethanol (90% and 100%), cleared in xylene using an automatic Leica autostainer XL, and finally cover-slipped with Cytoseal 60 (Thermo Scientific: 8310-4) mounting medium. The other tissues harvested for the study were processed in a similar manner.

### 4.4. RNA Extraction and RNA Sequencing

The library construction and RNA-seq was carried out by Novogene Corporation Inc. (Sacramento, CA, USA). The library concentration was measured with a Qubit 2.0 fluorometer (Life Technologies, Carlsbad, CA, USA) and then diluted to 1 ng/µL. The insert size was assessed using an Agilent 2100, Santa Clara, CA, USA, followed by more precise quantification through quantitative PCR (qPCR), ensuring a library activity above 2 nM. Twenty million raw reads per sample were generated using paired-end (PE) 150 bp sequencing on the Illumina HiSeq platform.

### 4.5. Pathway and GO Term Enrichment Analyses

Pathway analyses were performed using the package clusterProfiler [64] v.4.4 for enrichment analysis, including GO terms curated in the gene ontology resource (https://geneontology.org, accessed on 14 May 2024), such as “biological process”, “molecular function”, and “cellular component”, using the KEGG (http://www.kegg.jp/, accessed on 14 May 2024) and Reactome database (http://www.reactome.org, accessed on 14 May 2024). Gene Set Enrichment Analysis (GSEA) was conducted to compare the query genes among the four diet groups. For this analysis, we utilized the Bioconductor package msigdb v.7.5.1. Significant enrichment was determined by considering an adjusted *p*-value (Padj) < 0.05.

### 4.6. Heat Map Generation

Heat maps were created using genes identified as differentially expressed with *q* < 0.05. Rows were centered and scaled using *z*-scores. The hierarchical clusters were created using Ward’s linkage method. Gene expression levels were normalized using log transformation to reduce skewness and ensure comparability across samples. The clustering was performed using the functions in R.

### 4.7. Principal Component Analysis

Principal component analysis (PCA) was performed on the normalized, log2-transformed gene expression data to identify underlying patterns and reduce dimensionality. The analysis was conducted using the prcomp function from the stats package in R.

### 4.8. Differential Expression Analyses

Differential expression analyses among the four diet groups were performed using the DESeq2 package (1.42.1) [65]. DESeq2 provides statistical routines for determining differential expression in digital gene expression data using a model based on the negative binomial distribution. The resulting *p*-values were adjusted using the Benjamini and Hochberg approach for controlling the false discovery rate (fdr). Genes with *q* < 0.05 found by DESeq2 and Log2 (Fold-change) of 1 were set as the thresholds for significant differential expression were assigned as differentially expressed. Further, to quantify gene expression levels, we employed Fragments Per Kilobase of transcript per Million mapped reads (FPKM). The DEG list generated includes genes with FPKM values greater than 1.

### 4.9. Statistical Analyses

To determine the statistical significance related to diet and age, an analysis of variance (ANOVA) was performed across the four dietary groups (SDF, SDGF, SDGM, and SDM) using the aov function in R [66]. The weight was used as the dependent variable, and the group was the independent variable. Since the ANOVA test revealed a significant difference in weight between groups, a Tukey’s Honest Significant Difference (HSD) test was conducted using the TukeyHSD function from the rstatix package [67] to identify which specific groups differed from each other. This test employed a family-wise confidence level of 95%. Values of *p* < 0.05 were considered significant, and detailed values of statistical significance are provided in figure legends and text. For survival analysis, the log-rank test was conducted between the two diet groups using R, considering *p* < 0.05 as significant. Chi-square (*χ*^2^) statistics and components (O-E)^2^/E and (O-E)^2^/V were used to assess statistical significance. Additional statistical methods are described in the text; data are shown as means ± SD.

## 5. Conclusions

Under scrupulous environmental conditions, the longevity of male or female C57BL/6J mice is unaffected by the sole addition of grapes to the diet. Notably, however, whereas no overt differences were observed with the liver, colon, muscle, prostate or ovary, the kidney maintenance of both genders was improved. The complete mechanism of this response remains to be elucidated. Our work shows a strong divergence of the phenotypic expression in the kidneys of males versus females, which is somewhat normalized when grapes are added to the diet. The detailed analyses of the alteration of specific gene expression, as well as the presumptive pathways thereby affected, are consistent with the promotion of improved kidney health. It would be of interest to know if these grape-induced responses are capable of affecting health- or life-spans under free-living conditions wherein the host is subject to typical environmental and dietary conditions; Table S7: Genes showing differences between SDGM and SDM (Figure 8A) but showing no difference for female groups.

## Figures and Tables

**Table 1:** Organ weight and organ-weight-to-body-weight ratios of 912-day-old male and female mice with or without a grape diet.

Weight or Length	SDM (*n =* 5)	SDGM (*n =* 5)	*p*-Value *	SDF (*n =* 5)	SDGF (*n =* 5)	*p*-Value *
Body weight (BW, g)	38.1 ± 3.0	44.7 ± 6.2	0.062	25.3 ± 4.4	29.3 ± 5.6	0.23
Liver (g)	2.86 ± 0.48	2.73 ± 0.62	0.73	1.76 ± 0.89	1.64 ± 0.34	0.75
Liver/BW ratio	0.075 ± 0.011	0.062 ± 0.017	0.18	0.067 ± 0.031	0.059 ± 0.013	0.44
Colon length (cm)	9.1 ± 0.8	9.5 ± 0.7	0.46	8.9 ± 1.4	8.9 ± 1.0	1.00
Colon (g)	0.222 ± 0.032	0.201 ± 0.051	0.62	0.204 ± 0.058	0.248 ± 0.042	0.20
Colon/BW ratio	0.006 ± 0.001	0.005 ± 0.001	0.12	0.008 ± 0.002	0.009 ± 0.001	0.55
Kidney (g)	0.522 ± 0.046	0.525 ± 0.037	0.93	0.494 ± 0.110	0.413 ± 0.095	0.25
Kidney/BW ratio	0.014 ± 0.001	0.012 ± 0.001	0.01	0.020 ± 0.045	0.014 ± 0.002	0.03
Prostate or ovary	0.328 ± 0.170	0.545 ± 0.290	0.19	0.0698 ± 0.053	0.0838 ± 0.068	0.73
Prostate/BW ratio	0.010 ± 0.005	0.0010 ± 0.004	0.91	NA **	NA **	NA **
Ovary/BW ratio	NA **	NA **	NA **	0.003 ± 0.002	0.003 ± 0.003	0.76
Hip muscle (g)	0.53 ± 0.13	0.63 ± 0.15	0.27	0.46 ± 0.16	0.46 ± 0.68	0.92
Hip muscle/BW ratio	0.013 ± 0.003	0.014 ± 0.004	0.85	0.050 ± 0.071	0.046 ± 0.067	0.93

* p-Values are determined between groups of the same sex (*n* = 5). ** NA indicates not applicable.

**Table 2:** Blood chemistry values of 912-day-old male and female mice with or without a grape diet.

	Male				Female			
Substance	SDM (*n =* 5)	SDGM (*n =* 5)	*p*-Value	Reference *	SDF (*n =* 5)	SDGF (*n =* 4)	*p*-Value	Reference *
ALB (g/dL)	3.46 ± 0.57	3.88 ± 0.30	0.18	2.8–3.8 (3.2)	3.44 ± 0.84	3.30 ± 0.68	0.79	2.4–4.3 (3.4)
ALP (U/L)	85.2 ± 42.3	91.6 ± 27.1	0.78	111–275 (195)	35.0 ± 16.4	28.0 ± 12.3	0.50	105–370 (95)
ALT (U/L)	241.8 ± 157.1	121.0 ± 56.9	0.14	28–129 (68)	51.2 ± 25.7	47.2 ± 17.8	0.80	27–195 (57)
AMY (U/L)	1016 ± 95	1057 ± 242	0.73	NA **	1613 ± 263	1396 ± 185	0.21	NA **
TBIL (mg/dL)	0.32 ± 0.045	0.32 ± 0.045	1.00	0.2–0.6 (0.3)	0.32 ± 0.45	0.375 ± 0.15	0.46	0.2–0.6 (0.3)
BUN (mg/dL)	42.6 ± 25.2	24.2 ± 9.1	0.16	7–28 (14)	55.0 ± 51.6	20.2 ± 5.4	0.23	5–26 (14)
Ca (mg/dL)	10.9 ± 0.31	11.0 ± 0.48	0.76	9.7–12.5 (11.0)	10.9 ± 0.78	10.7 ± 0.24	0.58	9.7–12.3 (11.1)
PHOS (mg/dL)	7.6 ± 4.0	9.4 ± 1.5	0.37	7.9–14.5 (11.1)	12.1 ± 4.7	9.0 ± 1.5	0.24	7.3–13.5 (10.5)
CRE (mg/dL)	0.34 ± 0.17	0.38 ± 0.18	0.72	0.2–0.5 (0.3)	0.36 ± 0.21	0.50 ± 0.29	0.43	0.2–0.5 (0.3)
GLU (mg/dL)	153.2 ± 48.9	195.0 ± 43.2	0.19	172–372 (259)	88.2 ± 47.2	124 ± 41.0	0.27	177–348 (240)
Na (mmol/L)	159.8 ± 2.5	158.0 ± 3.6	0.39	145–176 (158)	162.2 ± 5.6	155.0 ± 1.4	0.043	147–181 (159)
K (mmol/L)	6.88 ± 0.41	7.06 ± 0.46	0.53	7.6–11.2 (9.4)	7.24 ± 0.78	6.73 ± 0.30	0.25	7.3–11.8 (8.8)
TPR (g/dL)	5.86 ± 0.56	6.12 ± 0.23	0.36	4.8–7.0 (5.6)	5.76 ± 0.56	5.43 ± 1.3	0.62	4.8–7.2 (5.7)
GLOB (g/dL)	2.42 ± 0.26	2.20 ± 0.39	0.32	NA **	2.30 ± 0.49	2.15 ± 0.77	0.73	NA **

* Taken from https://www.criver.com/sites/default/files/resources/doc_a/C57BL6MouseModelInformationSheet.pdf (assessed on 15 April 2024). The range given is the 95% confidence interval followed by the mean value in parentheses. ** NA, not available.

**Table 3:** List of proinflammatory genes consisting of chemokines, interleukins, cytokines, and immune receptors comparing SDGF vs. SDF and SDGM vs. SDM in the kidney.

Gene Name	Padj.	Log2 (FoldChange)	SDGF_fpkm	SDF_fpkm	Sex
*Cxcl13*	<0.001	4.013	195.847	12.281	Female
*Ccl4*	<0.001	4.017	17.779	1.103	Female
*Ccl3*	0.001	2.877	4.646	0.634	Female
*Il10*	0.004	2.876	1.910	0.259	Female
*Cxcl13*	<0.001	−2.940	4.815	35.901	Male
*Ccl6*	0.001	−2.013	16.040	62.831	Male
*Ccl8*	0.021	−1.714	22.995	73.282	Male
*Ccl24*	0.050	−2.635	0.285	1.750	Male

**Table 4:** Dietary constituents: 4% fat standard diet (SD; TD.160157); SD + 5% (*w*/*w*) standardized grape powder (SDG; TD.160158); diets were produced by Envigo (Madison, WI, USA).

	Standard Diet (TD.160157) ^3^	Standard Diet with 5% Grape Powder (TD.160158) ^3^
**Formula (g/kg)**		
Casein	195	192.9
DL-Methionine	3.0	3.0
Sucrose	191.1	191.0
Dextrose, anhydrous	66.45	44.3
Fructose	66.45	44.3
Corn starch	235.03	232.88
Maltodextrin	100	100.0
Anhydrous milk fat ^1^	30	29.85
Soybean oil	10	10
Cellulose	50	50
Mineral mix, AIN-76 (170915)	35	35
Potassium citrate, monohydrate	4.03	2.69
Calcium carbonate	4.0	4.0
Vitamin mix, Teklad (40060)	10.0	10.0
Ethoxyquin, antioxidant	0.04	0.04
Grape powder, freeze-dried ^2^	0	50

^1^ For each 100 g: total fat, 99.8 g; saturated fat, 67 g; trans fat, 2.6 g; polyunsaturated fat, 3.9 g; monounsaturated fat, 26.3 g. ^2^ Grape powder is considered to contain 3.71 kcal/g, 3% fat, 88.6% carbohydrate (as a 1:1 mixture of fructose and glucose), 3.58% protein and 9.73 g/kg K^+^. ^3^ Formulated to 3.6 Kcal/g (protein, 19.1%; carbohydrate, 70.5%; fat, 10.4%).

## Data Availability

The datasets generated and analyzed for the current study are available in the National Center for Biotechnology Information (NCBI) repository. Bioproject accession number PRJNA1113666.

## Supplementary Materials

The following supporting information can be downloaded at: https://www.mdpi.com/article/10.3390/nu16142309/s1, Table S1: Comparison of the body weight of SDM vs. SDGM based on survival percentiage. The analysis was conducted using the Mann–Whitney test where Wilcoxon rank sum test (W) and resulting *p*-values were generated as summarized in the table; Table S2: Comparison of the body weight of SDF vs. SDGF based on survival percentage. The analysis was conducted using the Mann–Whitney test where Wilcoxon rank sum test (W) and resulting *p*-values were generated as summarized in the table; Table S3: List of proinflammatory genes consisting of chemokines, interleukins, cytokines, and immune receptors comparing SDGF vs. SDF in kidney. Those showing statistically significant differences are listed in Table 3; Table S4: List of proinflammatory genes consisting of chemokines, interleukins, cytokines, and immune receptors comparing SDGM vs. SDM in kidney. Those showing statistically significant differences are listed in Table 3; Table S5: List of proinflammatory genes consisting of chemokines, interleukins, cytokines, and immune receptors comparing SDGM vs. SDM in liver. Table S6: List of proinflammatory genes consisting of chemokines, interleukins, cytokines, and immune receptors comparing SDGF vs. SDF in liver; Table S8: Genes showing differences between SDGF and SDF (Figure 8B) but showing no difference for male groups.

## Abbreviations

ALB, albumin; ALP, alkaline phosphatase; ALT, alanine aminotransferase; AKI, acute kidney injury; AMY, amylase; ANOVA, analysis of variance; BUN, blood urea nitrogen; CKD, chronic kidney disease; CRE, creatinine; DEG, differential expressed gene; ECM, extracellular matrix; FcγRs, Fc-gamma receptors; FGA, fibrinogen Aα chain; GLOB, globulin; FPKM, Fragments Per Kilobase of transcript per Million mapped reads; Glu, glucose; GSEA, gene set enrichment analysis; H&E, hematoxylin and eosin; HSD, Honest Significant Difference; IACUC, Institutional Animal Care and Use Committee; KEGG, Kyoto Encyclopedia of Genes and Genomes; PE, paired-end; PID, pathway interactive database; PCA, principal component analysis; PHOS, phosphorus; qPCR, quantitative Polymerase Chain Reaction; RFID, radio-frequency identification; ROS, reactive oxygen species; SDF, standard diet females; SDGF, standard diet containing 5% grape powder females; SDGM, standard diet containing 5% grape powder males; SDM, standard diet males; TLR, Toll-like receptor; TGF-β, transforming growth factor-β; TCA, tricarboxylic acid cycle; TBIL, total bilirubin; TP, total protein.
