# Supplementary material for: Long-Term Dietary Consumption of Grapes Affects Kidney Health in C57BL/6J Mice
Source: Nutrients. 2024 Jul 18;16(14):2309. doi: 10.3390/nu16142309 (PMC11280382; doi:10.3390/nu16142309)
Supplement: Supplementary file 1 [file nutrients-16-02309-s001.zip › nutrients-3072860-supplementary.pdf]

Supplementary Data

Table S1. Comparison of the body weight of SDM vs. SDGM based on survival percentage. The analysis was conducted using the Mann–Whitney test where Wilcoxon rank sum test (W) and resulting *p*-values were generated as summarized in the table.

Survival of Mice	W	*p* Value
25 percent dead	114	0.130
75 percent survived	2511	0.101
50 percent dead	444	0.101
50 percent survived	1097	0.052
75 percent dead	1568	0.022
25 percent survived	199	0.321
100 percent dead	4511	0.232

Table S2. Comparison of the body weight of SDF vs. SDGF based on survival percentage. The analysis was conducted using the Mann–Whitney test where Wilcoxon rank sum test (W) and resulting *p*-values were generated as summarized in the table.

Survival of Mice	W	*p* Value
25 percent dead	161	0.583
75 percent survived	2366	0.529
50 percent dead	712	0.633
50 percent survived	1535	0.850
75 percent dead	2228	0.716
25 percent survived	270	0.418
100 percent dead	4919	0.844

Table S3. List of proinflammatory genes consisting of chemokines, interleukins, cytokines, and immune receptors comparing SDGF vs. SDF in kidney. Those showing statistically significant differences are listed in Table 3.

Gene Name	Padj	Log2(FoldChange)	SDGF_fpkm	SDF_fpkm
Cxcl9	0.462	1.186	4.591	2.044
C730027H18Rik	0.615	2.485	0.639	0.111
Lepr	0.638	−1.036	2.383	4.950
Cd40lg	0.704	−2.525	0.089	0.544
Tnfrsf19	0.751	−1.131	0.516	1.147
Tnfrsf18	0.878	−1.282	0.657	1.624
Il1b	0.917	1.093	2.362	1.120
Il10ra	0.953	0.903	6.327	3.427
Tnfrsf25	0.953	1.129	1.246	0.576
Ccl1	0.953	5.724	0.141	0.000
Ccr5	1.000	0.842	3.218	1.818
Ptp4a1	1.000	−1.547	0.070	0.210
Ccl7	1.000	1.085	3.055	1.454
Tnfsf10	1.000	−0.771	6.220	10.756
Il20rb	1.000	−0.978	0.410	0.819
5S_rRNA	1.000	5.467	2.418	0.000
U6	1.000	−5.257	0.000	2.247
5S_rRNA	1.000	−5.257	0.000	2.247
Ccl9	1.000	0.737	11.487	6.981

Il18	1.000	0.890	2.589	1.412
Osm	1.000	1.642	0.269	0.085
Edar	1.000	−0.972	0.214	0.427
1700030C10Rik	1.000	−4.944	0.000	0.132
Il19	1.000	5.153	0.194	0.000
Aldoa	1.000	1.454	0.835	0.304
Tnfrsf14	1.000	0.722	2.475	1.519
Gm16364	1.000	−1.345	0.157	0.411
Ccl2	1.000	0.601	4.984	3.327
Cxcr2	1.000	1.330	0.201	0.080
Ccl6	1.000	0.531	25.373	17.791
Ltb	1.000	−0.530	6.236	9.124
Cxcr5	1.000	−0.631	0.751	1.180
Tmem147os	1.000	0.691	1.532	0.960
Cxcl11	1.000	0.979	0.176	0.090
Cxcl12	1.000	−0.479	27.969	39.487
5S_rRNA	1.000	−1.987	0.551	2.403
Il18bp	1.000	−0.524	2.224	3.240
Cxcr6	1.000	−0.540	2.218	3.267
Tnfrsf8	1.000	−0.838	0.193	0.351
Il18r1	1.000	−0.514	1.420	2.055
Il1rl2	1.000	−0.543	0.531	0.784
U6	1.000	−4.545	0.000	1.655
Tmem147os	1.000	4.751	0.366	0.000
U6	1.000	−4.545	0.000	1.374
Il33	1.000	−0.457	13.219	18.381
Ccl20	1.000	0.734	0.900	0.546
Tnfsf14	1.000	0.600	1.024	0.683
C730027H18Rik	1.000	0.555	0.587	0.404
Ccl8	1.000	0.427	28.730	21.655
Tnfrsf11a	1.000	0.459	1.254	0.924
Osmr	1.000	−0.405	3.590	4.816
Il31ra	1.000	0.757	0.186	0.111
7SK	1.000	0.790	2.617	1.523
Gm35558	1.000	−0.993	0.150	0.306
Tgfbr3	1.000	−0.392	9.151	12.169
Cntfr	1.000	−0.405	5.057	6.786
Ccl12	1.000	0.483	4.867	3.525
Cxcl10	1.000	0.390	6.470	5.000
7SK	1.000	−1.674	0.169	0.588
Cxcl15	1.000	−1.674	0.026	0.091
Ccl25	1.000	0.460	0.381	0.280
Tnfsf12	1.000	0.361	12.554	9.899
Ccl11	1.000	0.402	3.803	2.916
Tnfrsf4	1.000	0.508	1.100	0.782
Il1r1	1.000	−0.326	2.421	3.074
Lif	1.000	0.360	1.202	0.948
St6galnac2	1.000	−0.568	0.363	0.548
Cxcl1	1.000	−0.369	1.305	1.708
Ccl22	1.000	0.423	0.885	0.668
Cxcl2	1.000	0.587	0.350	0.235
Tnfsf8	1.000	−0.398	0.467	0.625

U6	1.000	4.193	1.171	0.000
U6	1.000	−3.990	0.000	1.449
7SK	1.000	4.193	0.340	0.000
Snhg4	1.000	4.193	0.051	0.000
Gm5468	1.000	4.193	0.019	0.000
Gm35558	1.000	−3.990	0.000	0.195
U6	1.000	−3.990	0.000	0.891
7SK	1.000	4.193	0.415	0.000
U6	1.000	4.193	1.135	0.000
7SK	1.000	4.193	0.386	0.000
5S_rRNA	1.000	4.193	0.959	0.000
U6	1.000	4.193	1.011	0.000
Gm5468	1.000	−3.990	0.000	0.135
5S_rRNA	1.000	4.193	1.112	0.000
Lifr	1.000	−0.268	5.346	6.522
Ccl5	1.000	0.274	20.333	17.037
Cd40	1.000	−0.313	0.849	1.069
Il6	1.000	1.180	0.137	0.060
Ifngr1	1.000	−0.241	24.866	29.782
Cxcr3	1.000	−0.270	2.891	3.533
Pglyrp1	1.000	−0.289	1.302	1.612
Cx3cl1	1.000	−0.230	32.701	38.864
Snhg4	1.000	0.266	1.025	0.863
Gm16364	1.000	−0.768	0.169	0.296
Xcl1	1.000	−0.578	0.425	0.650
Cxcl5	1.000	0.334	0.746	0.599
Tgfbr1	1.000	0.195	11.294	9.998
Ifngr2	1.000	0.167	9.109	8.220
Tgfbr2	1.000	−0.163	17.654	20.019
Tnfsf13	1.000	−0.278	0.747	0.919
Ccr2	1.000	0.164	3.422	3.094
5S_rRNA	1.000	1.163	1.951	0.851
7SK	1.000	−0.751	0.404	0.706
Atf2	1.000	0.153	6.188	5.638
Gvin-ps1	1.000	−0.479	0.061	0.087
Tnfrsf11b	1.000	0.263	0.592	0.499
Cxcl14	1.000	−0.140	33.208	37.084
Ifnar1	1.000	−0.140	7.031	7.852
Ptp4a1	1.000	0.260	0.429	0.363
Il1a	1.000	−0.505	0.085	0.123
Il11	1.000	0.319	0.374	0.303
Sigirr	1.000	0.129	16.064	14.881
Il1rl1	1.000	0.174	0.282	0.253
Ifnar2	1.000	0.126	8.920	8.281
Tnfsf11	1.000	0.780	0.149	0.087
Aldoa	1.000	0.114	315.765	295.572
Ltbr	1.000	−0.116	28.750	31.566
Tnfsf15	1.000	−0.135	0.973	1.083
Tnfrsf17	1.000	−0.454	0.341	0.476
Il10rb	1.000	−0.109	8.222	8.985
1700030C10Rik	1.000	−0.243	0.513	0.615
Il1r2	1.000	0.214	0.583	0.509

Cxcl17	1.000	0.144	1.462	1.339
Cxcr4	1.000	0.091	5.137	4.886
Ccl24	1.000	−0.365	0.344	0.450
Ifng	1.000	0.213	0.429	0.375
Tnfrsf12a	1.000	0.079	24.092	23.111
Eda2r	1.000	0.102	0.463	0.437
Clcf1	1.000	−0.081	2.630	2.818
Il18rap	1.000	−0.113	0.387	0.424
St6galnac2	1.000	0.060	13.571	13.185
Ccl17	1.000	−0.170	1.407	1.605
Tnfrsf13b	1.000	−0.074	1.765	1.882
Tnfrsf21	1.000	0.034	102.276	101.214
Ccl28	1.000	−0.033	14.431	14.959
Ccr7	1.000	−0.081	0.984	1.055
Cxcl16	1.000	−0.024	22.108	22.781
Ccr1	1.000	0.032	1.453	1.439
Gvin-ps1	1.000	−0.193	0.307	0.358
Ccl19	1.000	−0.018	4.746	4.870
Ccr3	1.000	−0.352	0.034	0.044
Ccr6	1.000	−0.352	0.048	0.063
Tnfsf4	1.000	0.210	0.151	0.132
Ccr4	1.000	−0.104	0.072	0.078
Ccr8	1.000	0.203	0.095	0.083
U6	1.000	0.191	0.993	0.867
7SK	1.000	0.191	0.176	0.154
7SK	1.000	1.115	0.362	0.158
7SK	1.000	−0.721	0.197	0.343
7SK	1.000	0.191	0.185	0.162
7SK	1.000	−0.721	0.193	0.337
Cntf	1.000	−0.721	0.052	0.090
Cxcr1	1.000	0.191	0.037	0.033
Cd70	1.000	0.191	0.066	0.058
Cxcl3	1.000	1.115	0.032	0.014
5S_rRNA	1.000	1.115	0.897	0.391
5S_rRNA	1.000	0.191	0.448	0.391

Table S4. List of proinflammatory genes consisting of chemokines, interleukins, cytokines, and immune receptors comparing SDGM vs. SDM in kidney. Those showing statistically significant differences are listed in Table 3.

Gene Name	Padj	Log2(FoldChange)	SDGM_fpkm	SDM_fpkm
Ccl7	0.075	−2.553	0.461	2.671
Cxcr2	0.087	−2.792	0.081	0.558
Ccl9	0.091	−1.463	5.435	14.551
Cxcr5	0.153	−1.781	0.332	1.114
Ptp4a1	0.304	−1.935	0.134	0.501
Ccl17	0.304	−2.997	0.239	1.959
Tnfsf8	0.345	−1.686	0.226	0.711
Edar	0.364	2.758	0.254	0.035
7SK	0.374	3.359	2.685	0.229
Ccr1	0.447	−1.348	0.565	1.400
Tnfrsf4	0.447	−1.893	0.315	1.153
Il1r2	0.472	−2.441	0.097	0.528

Ptp4a1	0.560	−3.241	0.011	0.114
Il1rl2	0.576	−1.184	0.280	0.619
Il11	0.606	−2.586	0.059	0.363
Cxcl2	0.678	−1.536	0.268	0.761
Lif	0.693	−1.125	0.382	0.810
Cd40lg	0.693	−5.652	0.000	0.302
Cxcr4	0.709	−0.958	2.416	4.560
Cxcl5	0.724	−1.718	0.198	0.642
Ccl20	0.724	−1.815	0.216	0.753
Tnfrsf13b	0.726	−0.953	1.215	2.286
Cxcr6	0.766	−1.001	1.306	2.542
Il18	0.831	−1.071	1.253	2.564
Clcf1	0.831	−0.842	1.843	3.208
Osmr	0.856	−0.744	2.707	4.401
Ccl2	0.907	−0.804	4.109	6.968
Tnfsf10	0.907	0.710	13.937	8.270
Cxcl11	0.907	−2.734	0.016	0.116
Il33	0.946	−0.691	8.569	13.433
Tnfrsf21	0.946	0.675	88.409	53.746
Cxcr3	0.948	−0.894	1.066	1.927
Ifngr1	0.996	−0.649	18.886	28.738
Il1rl1	0.996	−0.911	0.170	0.310
Cntfr	0.996	−0.673	3.361	5.203
Ccr2	0.996	−0.660	1.893	2.905
Ccr5	0.996	−0.671	0.985	1.522
Tnfrsf11b	0.996	−0.842	0.589	1.027
Il18r1	0.996	−0.711	0.830	1.320
Ccl11	0.996	−0.704	4.890	7.736
7SK	0.996	−5.082	0.000	0.701
Cxcl1	1.000	−0.736	1.805	2.920
U6	1.000	−2.516	0.918	5.636
Il1b	1.000	−0.747	1.194	1.947
Tnfrsf8	1.000	−1.506	0.071	0.200
Tnfrsf18	1.000	−0.758	0.928	1.528
Tnfrsf12a	1.000	−0.547	16.813	23.845
Ifnar2	1.000	−0.510	6.209	8.583
Tnfsf14	1.000	−1.064	0.266	0.545
U6	1.000	−4.681	0.000	1.987
5S_rRNA	1.000	−4.681	0.000	1.587
7SK	1.000	4.608	0.564	0.000
Xcl1	1.000	1.044	1.294	0.602
Ccl19	1.000	−0.551	3.264	4.646
Il18bp	1.000	−0.518	1.301	1.809
C730027H18Rik	1.000	0.494	1.639	1.130
Il20rb	1.000	−0.857	0.161	0.284
Cxcl9	1.000	−0.477	1.652	2.233
Lepr	1.000	−0.420	2.522	3.277
Ccl12	1.000	−0.568	2.520	3.633
Tnfrsf25	1.000	−0.833	0.270	0.470
1700030C10Rik	1.000	−0.692	0.461	0.725
Il1r1	1.000	−0.401	1.798	2.305
Tnfrsf17	1.000	0.908	0.755	0.386

Il6	1.000	−1.263	0.076	0.181
Il10rb	1.000	−0.357	7.351	9.140
Ccl22	1.000	−0.804	0.238	0.406
Il10ra	1.000	−0.374	2.603	3.275
Ccl4	1.000	−0.746	0.932	1.526
Osm	1.000	0.904	0.217	0.111
Ccl28	1.000	−0.324	3.175	3.859
Pglyrp1	1.000	−0.415	1.005	1.302
Gm35558	1.000	0.758	0.250	0.142
Tgfbr2	1.000	−0.291	16.006	19.011
Il10	1.000	−1.343	0.094	0.240
7SK	1.000	−1.343	0.435	1.112
Ccl5	1.000	−0.303	11.663	13.964
Tgfbr3	1.000	−0.281	8.431	9.943
7SK	1.000	−4.124	0.000	0.381
5S_rRNA	1.000	4.052	1.218	0.000
5S_rRNA	1.000	−4.124	0.000	1.095
U6	1.000	4.052	1.139	0.000
U6	1.000	−4.124	0.000	1.187
Cntf	1.000	−4.124	0.000	0.117
Cxcr1	1.000	4.052	0.083	0.000
5S_rRNA	1.000	−4.124	0.000	1.165
Gm35558	1.000	4.052	0.247	0.000
U6	1.000	4.052	1.129	0.000
5S_rRNA	1.000	4.052	0.992	0.000
5S_rRNA	1.000	−4.124	0.000	1.085
U6	1.000	−4.124	0.000	1.210
7SK	1.000	4.052	0.405	0.000
Il22	1.000	−4.124	0.000	0.113
7SK	1.000	4.052	0.500	0.000
Eda2r	1.000	−0.396	0.251	0.321
Tnfsf15	1.000	−0.355	0.443	0.551
Cxcl17	1.000	0.897	0.485	0.248
Tnfrsf11a	1.000	0.316	0.647	0.505
Tmem147os	1.000	0.353	1.347	1.022
Il1a	1.000	1.404	0.093	0.032
1700030C10Rik	1.000	−1.544	0.042	0.128
Sigirr	1.000	0.192	24.563	20.868
Atf2	1.000	0.183	6.943	5.935
Cxcl10	1.000	−0.200	4.562	5.086
Cx3cl1	1.000	−0.166	31.308	34.094
Tnfsf11	1.000	−1.029	0.055	0.113
Ifnar1	1.000	−0.161	6.784	7.364
C730027H18Rik	1.000	−0.217	1.366	1.542
Ifngr2	1.000	−0.146	8.901	9.557
Cxcl12	1.000	−0.138	30.624	32.707
Tnfrsf14	1.000	−0.213	1.283	1.444
Aldoa	1.000	0.496	0.462	0.315
Tnfrsf19	1.000	−0.175	0.364	0.398
Tmem147os	1.000	0.401	0.944	0.690
Il18rap	1.000	−0.168	0.273	0.298
Tnfsf12	1.000	−0.111	9.746	10.219

Cxcl16	1.000	−0.103	20.486	21.354
Cd40	1.000	−0.161	0.885	0.961
St6galnac2	1.000	0.073	16.628	15.348
Cxcl14	1.000	−0.068	29.531	30.039
St6galnac2	1.000	0.129	0.548	0.486
Lifr	1.000	−0.039	5.686	5.671
Ltbr	1.000	−0.035	30.708	30.532
Ccl25	1.000	0.050	0.397	0.372
Ltb	1.000	−0.023	5.600	5.521
Aldoa	1.000	−0.018	320.829	315.265
Tnfsf13	1.000	−0.199	0.413	0.461
7SK	1.000	−0.189	2.895	3.209
Ccl3	1.000	−0.331	0.402	0.493
Il31ra	1.000	−0.628	0.022	0.033
7SK	1.000	0.484	0.615	0.420
Tgfbr1	1.000	0.001	8.681	8.418
Gvin-ps1	1.000	−0.075	0.127	0.129
Gvin-ps1	1.000	−0.987	0.113	0.232
Ccr7	1.000	0.076	0.933	0.859
Gm16364	1.000	−0.075	0.521	0.533
Gm16364	1.000	−0.987	0.047	0.096
Ccr3	1.000	−0.987	0.019	0.038
Ccr6	1.000	−0.067	0.027	0.027
Tnfsf4	1.000	−0.987	0.033	0.068
Ccr4	1.000	0.239	0.099	0.081
Ccr8	1.000	−0.387	0.209	0.268
Ifng	1.000	−0.073	0.143	0.146
Cxcl15	1.000	−0.067	0.029	0.030

Table S5. List of proinflammatory genes consisting of chemokines, interleukins, cytokines, and immune receptors comparing SDGM vs. SDM in liver.

Gene Name	Padj	Log2(FoldChange)	SDGM_fpkm	SDM_fpkm
Cxcl13	<0.001	−6.775	1.534	164.501
Ccl6	<0.001	−2.457	14.889	79.526
Il1b	0.001	−2.289	1.896	9.033
Cxcl14	0.001	−2.064	4.152	16.903
Lepr	0.002	−1.867	3.041	10.789
Pglyrp1	0.003	−2.887	0.336	2.459
U6	0.019	6.873	12.830	0.000
Tnfsf14	0.034	−2.899	0.143	1.081
Il10ra	0.037	−1.518	2.597	7.237
7SK	0.037	3.812	6.572	0.412
Ccl8	0.061	−2.150	1.553	6.783
Tnfrsf12a	0.072	−1.332	10.683	26.151
Ccr1	0.147	−1.448	0.934	2.484
Il6	0.156	−6.174	0.000	0.346
Il1a	0.166	−1.430	1.296	3.406
Tnfsf13	0.171	−3.581	0.054	0.706
Tnfrsf11a	0.183	−1.430	0.354	0.930
Ccl3	0.193	−2.653	0.345	2.194
Il18rap	0.217	−1.860	0.105	0.373

Ccl4	0.222	−1.494	2.138	5.889
Cxcr4	0.247	−1.150	3.415	7.378
Cx3cl1	0.282	−1.163	1.190	2.593
Ccl20	0.298	1.804	1.614	0.444
Il10rb	0.343	−0.996	3.057	5.931
Cxcr2	0.347	−1.668	0.232	0.724
Cxcl11	0.350	1.425	1.000	0.360
Il18bp	0.361	−0.945	7.841	14.683
Cxcr6	0.391	−1.330	0.731	1.795
Ccl12	0.424	−5.765	0.000	1.174
Ccr3	0.424	−5.765	0.000	0.193
7SK	0.424	5.697	2.058	0.000
Ccr2	0.479	−0.877	1.540	2.750
Gvin-ps1	0.513	−2.937	0.012	0.101
Il18r1	0.577	−0.957	0.623	1.178
7SK	0.577	5.222	1.396	0.000
Ccl7	0.607	−1.084	2.092	4.327
Il1r2	0.637	−1.530	0.231	0.661
Ifngr1	0.645	−0.706	13.818	21.916
Cxcl16	0.659	−0.707	7.358	11.683
Lifr	0.671	0.680	16.460	9.991
Tnfrsf14	0.692	−0.811	3.251	5.551
Il10	0.736	−1.831	0.202	0.722
Tnfsf10	0.736	0.669	3.752	2.294
Gvin-ps1	0.736	−5.194	0.000	0.774
Osm	0.736	−5.194	0.000	0.185
7SK	0.736	−5.194	0.000	1.405
Tnfsf8	0.740	−2.027	0.046	0.193
U6	0.740	−2.027	1.664	6.937
5S_rRNA	0.754	2.171	4.642	0.921
Ccl9	0.761	−0.597	25.143	36.979
Tnfrsf19	0.761	0.662	2.960	1.818
Tnfrsf21	0.778	−0.607	5.903	8.743
Ccr5	0.818	−0.616	1.537	2.292
Ccl19	0.823	0.871	2.747	1.454
Il33	0.852	−0.659	1.895	2.912
Tnfrsf13b	0.893	−0.705	0.840	1.333
Cxcr3	0.893	−0.733	1.274	2.063
Ptp4a1	0.893	0.670	0.653	0.398
Tnfsf11	0.893	4.511	0.118	0.000
Edar	0.893	−4.792	0.000	0.087
7SK	0.893	4.511	0.819	0.000
U6	0.893	−4.792	0.000	2.945
7SK	0.905	−1.473	0.802	2.228
Ccl2	0.906	−0.504	8.836	12.184
Ccl24	0.949	−0.593	2.043	3.001
Ifngr2	0.974	−0.418	12.129	15.755
Eda2r	0.975	−0.529	0.584	0.820
Cd40	0.989	−0.568	0.855	1.234
Cxcl5	0.989	−0.866	0.379	0.677
Cxcl2	0.989	−0.866	0.341	0.610
Il1r1	0.989	−0.385	10.715	13.602

Tgfbr2	0.989	−0.363	11.420	14.280
Cxcl9	0.989	0.364	17.284	13.060
Cntfr	0.989	−0.429	1.902	2.492
Osmr	0.989	−0.382	1.027	1.301
Clcf1	0.989	−0.457	0.950	1.270
Gm35558	0.989	−1.074	0.159	0.331
Tnfrsf11b	0.989	0.409	2.191	1.603
Sigirr	0.989	−0.324	5.275	6.420
Ccl11	0.989	0.990	0.408	0.194
Ccl25	0.989	0.353	0.853	0.649
Il31ra	0.989	3.957	0.031	0.000
Ccr8	0.989	3.957	0.150	0.000
U6	0.989	3.957	3.150	0.000
U6	0.989	3.957	2.051	0.000
5S_rRNA	0.989	3.957	1.534	0.000
7SK	0.989	3.957	0.623	0.000
Ccl1	0.989	−4.233	0.000	0.089
Gm35558	0.989	3.957	0.354	0.000
5S_rRNA	0.989	3.957	1.423	0.000
Ccl26	0.989	−4.233	0.000	0.745
7SK	0.989	3.957	0.500	0.000
7SK	0.989	3.957	0.600	0.000
U6	0.989	3.957	3.267	0.000
7SK	0.989	3.957	0.639	0.000
Ptp4a1	1.000	0.334	1.085	0.836
Cxcl10	1.000	−0.233	10.575	12.080
Tnfrsf4	1.000	0.859	0.582	0.308
Cxcr5	1.000	−0.291	0.688	0.820
Ccl17	1.000	1.203	0.515	0.204
Tnfsf12	1.000	−0.184	10.480	11.579
Lif	1.000	−0.439	0.182	0.241
Ccl28	1.000	0.403	0.237	0.173
Ifnar1	1.000	−0.141	10.261	10.999
Ltb	1.000	−0.161	5.993	6.513
Il20rb	1.000	−0.396	0.273	0.350
Il11	1.000	−0.286	0.212	0.252
Atf2	1.000	0.121	5.785	5.171
Il18	1.000	−0.130	8.812	9.373
C730027H18Rik	1.000	−0.118	2.008	2.119
Ifnar2	1.000	−0.103	22.150	23.128
Ccl5	1.000	−0.100	30.136	31.402
Tmem147os	1.000	−0.289	0.623	0.742
C730027H18Rik	1.000	−0.082	3.985	4.102
Cxcl1	1.000	0.048	29.542	27.786
Tgfbr1	1.000	0.046	8.572	8.070
Tgfbr3	1.000	0.044	3.234	3.049
Ccr7	1.000	−0.100	1.427	1.487
Cxcl12	1.000	0.033	63.207	60.048
Ltbr	1.000	0.030	78.386	74.652
Tnfrsf8	1.000	0.378	0.204	0.152
Il1rl2	1.000	−0.044	0.246	0.247
Xcl1	1.000	0.278	0.506	0.402

Il1rl1	1.000	0.650	0.026	0.015
Tnfrsf18	1.000	−0.081	1.273	1.310
Tnfsf15	1.000	−0.275	0.030	0.036
Tnfrsf25	1.000	−0.281	0.116	0.138
Ccl22	1.000	−0.017	0.873	0.858
7SK	1.000	0.650	0.491	0.293
Tnfsf4	1.000	−1.185	0.048	0.114
Cd40lg	1.000	0.650	0.141	0.084
Ccr4	1.000	−1.185	0.029	0.068
Ifng	1.000	−1.185	0.068	0.162
U6	1.000	−0.275	2.005	2.387
Tmem147os	1.000	−1.185	0.193	0.461
U6	1.000	−0.260	0.817	0.973
7SK	1.000	−0.275	0.820	0.977
7SK	1.000	−0.275	0.525	0.625

Table S6. List of proinflammatory genes consisting of chemokines, interleukins, cytokines, and immune receptors comparing SDGF vs. SDF in liver.

Gene Name	Padj	Log2(FoldChange)	SDGF_fpkm	SDF_fpkm
Cxcl14	0.001	2.151	14.267	3.270
Ccl4	0.006	2.516	9.549	1.688
Lepr	0.009	−1.712	13.456	44.938
Cxcl13	0.019	1.638	73.953	24.217
Ccr5	0.049	1.555	3.533	1.225
Tnfsf14	0.141	3.102	0.969	0.110
Il1b	0.147	1.436	7.059	2.655
Ccl24	0.206	−1.431	2.584	7.116
Tnfrsf13b	0.223	1.517	2.113	0.750
Ccl8	0.260	−1.635	2.741	8.720
C730027H18Rik	0.260	1.614	0.792	0.262
Il1rl1	0.322	2.829	0.214	0.029
Cxcl10	0.333	1.278	4.181	1.754
Ccr2	0.356	1.152	2.256	1.034
Tnfrsf4	0.356	−1.811	0.513	1.855
Il10ra	0.457	1.028	8.052	4.024
Ccl3	0.457	1.799	2.743	0.793
Ccr3	0.460	2.669	0.404	0.062
Ccl7	0.460	5.859	0.777	0.000
Ccl25	0.509	1.399	0.460	0.177
Tnfrsf11a	0.581	1.141	1.027	0.474
Cxcl5	0.640	2.172	0.501	0.109
Ccl22	0.735	−1.281	0.402	1.002
Cxcl1	0.754	0.873	9.420	5.239
Il20rb	0.777	−1.527	0.129	0.385
Tnfrsf12a	0.777	−0.792	11.518	20.338
Tmem147os	0.777	1.336	1.429	0.572
Tnfrsf8	0.777	−1.417	0.202	0.556
Il31ra	0.777	5.453	0.082	0.000
Tmem147os	0.777	5.196	0.853	0.000
7SK	0.777	−5.540	0.000	2.355
Il10rb	0.814	0.781	4.843	2.872

Tgfbr3	0.820	0.748	5.635	3.419
Ccr6	0.879	−1.338	0.268	0.698
Tnfsf10	0.905	0.735	3.487	2.135
Clcf1	0.938	0.898	1.268	0.692
Il1r1	0.955	−0.617	19.431	30.367
Cxcr2	0.955	0.964	0.893	0.465
5S_rRNA	0.955	4.884	2.376	0.000
Lifr	0.975	−0.585	109.761	167.796
Ccl2	1.000	0.804	2.598	1.513
Il1a	1.000	0.736	1.931	1.180
Tnfrsf25	1.000	−1.722	0.103	0.356
Il18r1	1.000	0.823	0.549	0.315
Ccr1	1.000	0.788	1.154	0.679
Lif	1.000	−1.866	0.036	0.140
Tnfrsf14	1.000	0.595	5.952	4.014
Ptp4a1	1.000	0.835	0.449	0.256
Il20ra	1.000	4.485	0.096	0.000
Cd40lg	1.000	−4.824	0.000	0.243
U1	1.000	−4.824	0.000	1.923
Gm35558	1.000	4.485	1.144	0.000
Ccl1	1.000	4.485	0.099	0.000
7SK	1.000	4.485	0.849	0.000
5S_rRNA	1.000	−4.824	0.000	2.450
5S_rRNA	1.000	4.485	1.995	0.000
Ptp4a1	1.000	0.519	2.027	1.441
Il18	1.000	0.510	9.887	7.072
Il10	1.000	−0.873	0.535	1.005
Il33	1.000	0.492	2.858	2.070
Osm	1.000	−1.011	0.171	0.357
Tnfrsf17	1.000	1.406	0.668	0.249
Cntfr	1.000	0.454	3.524	2.621
Cxcr3	1.000	−0.587	1.259	1.931
1700030C10Rik	1.000	0.830	0.718	0.409
Edar	1.000	1.036	0.171	0.084
U6	1.000	−0.914	3.276	6.396
Ccl17	1.000	−2.203	0.151	0.788
Il11	1.000	1.547	0.150	0.049
7SK	1.000	1.547	1.014	0.330
Tnfrsf19	1.000	0.389	3.268	2.543
Cxcl9	1.000	−0.353	9.726	12.659
Sigirr	1.000	−0.358	6.725	8.786
Ccl5	1.000	0.364	19.858	15.718
Cxcr5	1.000	0.471	0.911	0.669
7SK	1.000	1.203	2.334	1.013
Osmr	1.000	0.338	1.773	1.430
Ltbr	1.000	−0.310	62.737	79.286
Tnfrsf18	1.000	−0.583	0.868	1.329
Eda2r	1.000	0.493	0.475	0.344
Ccl9	1.000	−0.297	46.226	57.868
7SK	1.000	1.188	1.621	0.703
Tnfsf4	1.000	3.932	0.084	0.000
7SK	1.000	3.932	0.550	0.000

Cntf	1.000	−4.264	0.000	0.189
U1	1.000	−4.264	0.000	1.350
5S_rRNA	1.000	3.932	1.441	0.000
5S_rRNA	1.000	3.932	1.255	0.000
7SK	1.000	−4.264	0.000	0.619
7SK	1.000	3.932	0.540	0.000
U1	1.000	3.932	1.066	0.000
U6	1.000	3.932	1.511	0.000
7SK	1.000	3.932	0.622	0.000
Il1r2	1.000	−0.519	0.652	0.955
Tnfsf15	1.000	−0.665	0.107	0.174
Il18bp	1.000	−0.246	16.200	19.578
Tnfsf8	1.000	−0.442	0.307	0.426
Cxcl2	1.000	−0.602	0.188	0.294
Ifngr1	1.000	0.226	20.662	17.999
Tgfbr1	1.000	0.223	9.301	8.121
Tnfrsf21	1.000	0.223	9.146	7.985
Cxcl11	1.000	0.312	0.943	0.774
Cxcr4	1.000	0.251	4.906	4.201
C730027H18Rik	1.000	0.409	1.013	0.775
Ltb	1.000	−0.224	5.441	6.480
Ccr7	1.000	0.313	1.062	0.870
Pglyrp1	1.000	0.241	3.261	2.812
7SK	1.000	0.514	3.357	2.383
Cxcl16	1.000	0.185	7.778	6.973
Il18rap	1.000	0.305	0.346	0.285
Atf2	1.000	−0.158	4.600	5.232
Ifngr2	1.000	0.139	11.794	10.915
Tgfbr2	1.000	−0.132	10.521	11.751
Cd40	1.000	0.210	0.777	0.684
Cxcl12	1.000	0.109	58.871	55.639
Tnfrsf11b	1.000	0.126	2.347	2.192
Cxcr6	1.000	0.182	1.169	1.049
Il1rl2	1.000	−0.159	0.183	0.208
Ifnar2	1.000	−0.062	20.335	21.629
Ifnar1	1.000	0.060	9.327	9.121
Tnfsf12	1.000	−0.037	9.399	9.826
Ccl6	1.000	0.009	53.925	54.612
Ccl28	1.000	−0.128	0.112	0.125
Tnfsf13	1.000	−0.157	0.380	0.433
Gm35558	1.000	−0.088	0.210	0.228
U6	1.000	−0.088	4.244	4.603
Ccl19	1.000	0.087	1.705	1.635
Cx3cl1	1.000	0.022	0.693	0.696
Ccl12	1.000	−0.314	0.145	0.189
1700030C10Rik	1.000	−0.339	0.159	0.207
5S_rRNA	1.000	−0.332	1.365	1.777
Cxcr1	1.000	0.226	0.313	0.272
5S_rRNA	1.000	−0.314	0.654	0.851
7SK	1.000	−0.314	0.308	0.400
7SK	1.000	−0.332	0.659	0.858
7SK	1.000	0.595	0.482	0.314

7SK	1.000	−1.241	0.270	0.703
U6	1.000	−0.314	0.846	1.101

Table S7. Genes showing differences between SDGM and SDM (Figure 8A) but showing no difference for female groups.

Gene Names	Padj	SDGF - Log10(fpkm+1)	SDF - Log10(fpkm+1)
alox5	1.000	0.192	0.154
btk	0.157	0.532	0.267
fga	1.000	1.415	1.520
fpr1	0.943	0.284	0.124
hmox1	1.000	1.341	1.368
lox	0.739	0.859	1.128
ltf	0.375	0.225	0.083
lyve1	1.000	0.593	0.552
marco	1.000	0.250	0.280
mmp8	1.000	0.128	0.086
prg4	0.953	0.605	0.417
s100a8/9	0.427	1.090	0.760
vsig4	1.000	0.252	0.297

Table S8. Genes showing differences between SDGF and SDF (Figure 8B) but showing no difference for male groups.

Gene Names	Padj	SDGM - Log10(fpkm+1)	SDM - Log10(fpkm+1)
camp	0.845	0.000	0.151
cd300lf	0.804	0.067	0.143
cd72	1.000	0.516	0.633
fcgr4	1.000	0.890	1.032
fgr	1.000	0.411	0.374
fpr2	1.000	0.209	0.316
htra4	1.000	0.201	0.232
il10	1.000	0.039	0.093
lilrb4b	0.606	0.198	0.341
lipg	1.000	0.063	0.084
pilra	1.000	0.240	0.296
tlr8	0.326	0.155	0.334
